# Impaired reward prediction error encoding and striatal-midbrain connectivity in depression

**DOI:** 10.1038/s41386-018-0032-x

**Published:** 2018-02-26

**Authors:** Poornima Kumar, Franziska Goer, Laura Murray, Daniel G. Dillon, Miranda L. Beltzer, Andrew L. Cohen, Nancy H. Brooks, Diego A. Pizzagalli

**Affiliations:** 1 Center for Depression, Anxiety and Stress Research, McLean Hospital, Belmont, MA USA; 2 Department of Psychiatry, Harvard Medical School, Boston, MA USA; 3 McLean Imaging Center, McLean Hospital, Belmont, MA USA

## Abstract

Anhedonia (hyposensitivity to rewards) and negative bias (hypersensitivity to punishments) are core features of major depressive disorder (MDD), which could stem from abnormal reinforcement learning. Emerging evidence highlights blunted reward learning and reward prediction error (RPE) signaling in the striatum in MDD, although inconsistencies exist. Preclinical studies have clarified that ventral tegmental area (VTA) neurons encode RPE and habenular neurons encode punishment prediction error (PPE), which are then transmitted to the striatum and cortex to guide goal-directed behavior. However, few studies have probed striatal activation, and functional connectivity between VTA-striatum and VTA-habenula during reward and punishment learning respectively, in unmedicated MDD. To fill this gap, we acquired fMRI data from 25 unmedicated MDD and 26 healthy individuals during a monetary instrumental learning task and utilized a computational modeling approach to characterize underlying neural correlates of RPE and PPE. Relative to controls, MDD individuals showed impaired reward learning, blunted RPE signal in the striatum and overall reduced VTA-striatal connectivity to feedback. Critically, striatal RPE signal was increasingly blunted with more major depressive episodes (MDEs). No group differences emerged in PPE signals in the habenula and VTA or in connectivity between these regions. However, PPE signals in the habenula correlated positively with number of MDEs. These results highlight impaired reward learning, disrupted RPE signaling in the striatum (particularly among individuals with more lifetime MDEs) as well as reduced VTA-striatal connectivity in MDD. Collectively, these findings highlight reward-related learning deficits in MDD and their underlying pathophysiology.

## Introduction

Major depressive disorder (MDD) is a complex, heterogenous psychiatric disorder [1] and despite decades of research, its pathophysiology remains incompletely understood. Emerging evidence suggests that anhedonia (hyposensitivity to rewards) and negative bias (hypersensitivity to punishments), which are cardinal features of MDD [2, 3], might stem from disrupted learning about rewards and punishments, respectively, and adapt behavior accordingly. In this context, MDD could be described as a reinforcement learning (RL) disorder characterized by blunted reward but enhanced punishment learning. The main goal of the current study was to test these hypotheses using functional magnetic resonance imaging (fMRI) in conjunction with computational modeling.

Over the past decade, there has been a burgeoning interest in applying computational algorithms to dissect RL in healthy and psychiatric populations. Using these models, individual differences can be captured by tracking trial-by-trial variability in learning. Learning occurs when there is a deviation between the expected and actual outcome, quantified as a prediction error (PE). This PE is then used to update value estimates that support better prediction of future rewards. Non-human primate findings have shown that phasic firing of dopamine (DA) neurons in the ventral tegmental area (VTA) encodes reward prediction error (RPE). These midbrain DA RPE signals are then transmitted to the striatum and cortex and used to update stimulus-action values and guide goal-directed behavior [4, 5]. Consistent with this, human fMRI studies have described RPE signals in cortico-striatal circuits including the striatum, midbrain and prefrontal cortex [6, 7], and these signals are altered by manipulations that affect phasic DA signaling [8–10].

Although DA’s involvement in reward learning is strongly supported, there is conflicting evidence for its association with punishment learning [11]. Moreover, lateral habenula neurons have been found to fire during an unexpected punishment or omission of an expected reward [12]. This signal excites GABAergic cells in the tail of the VTA known as the rostromedial tegmental nucleus (RMTg; [13]), which then inhibits DA neurons in the VTA [12, 14] and thereby reduces DA levels in the striatum, eventually promoting active avoidance [15]. Similarly, habenular neurons are inhibited by an unexpected reward or non-punishing outcome [12], which disinhibits the VTA DA neurons [15] and increases DA concentration in the striatum [16], reinforcing the rewarding action. Complementing this, human studies have found punishment prediction error (PPE) signals in the habenula [17–19]. The VTA may thus be an intermediary, controlling both reward and punishment learning through its DA and GABA neurons, respectively. As fMRI cannot dissociate BOLD signal based on neurotransmitters, this might explain why the VTA is activated during both reward [6, 7] and punishment [17] learning in human fMRI studies.

Beyond neuromodulators, the existence of two discrete and opponent systems involved in reward and punishment learning has been heavily studied. A recent meta-analysis reported distinct systems were involved in encoding RPE and PPE signals. This included the striatum, frontal operculum, and midbrain in the reward system [6, 7], and insula, thalamus, and habenula in the punishment system [6].

Consistent with the hypothesis that MDD is characterized by reward dysfunction, prior fMRI studies in MDD have highlighted blunted RPE signals in the striatum [20–22] during learning, but intact signals in a non-learning context, where error signals are encoded, but potentially not used to update behaviors due to the lack of a learning component in the task used [23]. Moreover, several non-learning studies (especially in the emotional processing literature) have reported that MDD is associated with increased responsivity to punishment or negative stimuli [2, 24, 25]. Very few studies have investigated this in the context of learning in MDD. One study [26] found that MDD individuals displayed reduced reward and punishment learning rates, particularly with increasing anhedonia, whereas other studies [20, 27] reported an association between depression and oversensitivity to punishment in geriatric MDD. More recently, MDD has been linked to reduced associative value signals in the habenula during punishment learning [28].

An important limitation of prior studies is that most MDD participants showing altered RPE signals were on antidepressant medication (except [28]), which is known to affect the neural responses to reinforcers [22, 29]. Thus, observed PE signals could have been partly influenced by medication. To overcome these limitations, we examined RL in unmedicated individuals with MDD using a well-established instrumental learning task in conjunction with a Q-learning computational model and a region of interest (ROI) approach. We hypothesized that, relative to controls, the MDD group would show blunted RPE signals in the striatum and VTA, but potentiated PPE signals in the VTA, habenula and insula. In addition, owing to preclinical evidence and initial human data highlighting the role of VTA-striatal and VTA-habenula connectivity during reward [30–33] and punishment learning [12, 14], respectively, psychophysiological interaction (PPI) analyses were implemented to probe functional connectivity among these regions during delivery of rewards and punishments. We specifically hypothesized that MDD individuals would exhibit reduced VTA-striatal connectivity during reward and enhanced VTA-habenula connectivity during punishment learning.

## Materials and methods

### Participants

Twenty-six healthy controls and 28 unmedicated individuals with MDD recruited from the community were enrolled and screened using the Structured Clinical Interview for the DSM-IV (SCID; [34]) and Hamilton Depression Rating Scale (HDRS; [35]). All participants provided written informed consent. Participants were right-handed and reported no medical or neurological illnesses, no contraindications to MRI, no lifetime substance dependence and no substance abuse in the past year. Detailed inclusion and exclusion criteria are listed in the Supplement. In a separate session, participants completed an instrumental RL task whilst in the fMRI scanner, as well as the Beck Depression Inventory-II (BDI-II; [36]) and the Snaith Hamilton Pleasure Scale (SHPS; [37]) to assess depressive and anhedonic symptoms.

### Instrumental RL task

After a short practice outside the scanner, participants performed three runs of the RL task (adapted from [9]) with monetary outcomes, each time with new pairs of stimuli (Supplement Fig S1). During each run of 120 trials (40 gain, 40 loss, 40 neutral), participants were presented with one of three stimulus pairs (gain, loss or neutral), which were associated with 80%/20% probabilities of the following: Gain ($10/Nothing), Loss (Nothing/-$10), Neutral ($0/Nothing). On each trial, stimulus pairs were presented side-by-side (position counterbalanced across trials) and participants were asked to choose one of the two stimuli (Supplement).

### Behavioral analyses

#### Task performance

Participants’ choices for each trial were averaged across the three runs, resulting in a learning curve composed of 40 choice scores for each participant. Linear mixed-effects models with *Trials*, *Valence* and *Group* as factors, tested for group differences in reward and punishment learning.

#### Computational model (Q-Learning)

A standard Q-learning algorithm calculated the expected value of choices and PE based on individual’s choice and feedback history [38]. Moreover, we tested how well the RL model fitted the observed data relative to chance (Supplement).

### Functional imaging and analyses

For details on neuroimaging acquisition, processing and general linear model design, see Supplemental Methods. For each participant, the linear coefficient of RPE and PPE regressors were compared to 0 and the resulting contrast images were taken to conduct within-group *t*-tests. A whole-brain one-sample *t*-test and exploratory *Group* x *Valence* flexible factorial ANOVA were also run to explore brain regions showing *Group* and *Valence* effects.

#### ROI analyses

A staged ROI selection was implemented. Specifically, priority was given to clusters emerging from meta-analyses probing PE; for small regions hypothesized to be implicated in PE (VTA, habenula), clusters based on manual identification were used to avoid potential biases. Finally, for larger and functionally heterogenous regions (e.g., insula), a sphere was drawn around the coordinates emerging from prior studies using the same paradigm as used here. Based on these considerations, anatomically constrained bilateral striatum were extracted from a recent meta-analysis of RPE studies in healthy controls [7]. As prior studies have reported PPE signals in the right insula [9] and habenula [17–19], a right insula mask was created by drawing a sphere with 10 mm diameter around the peak voxel (40, 28, −6) reported in a prior study using a similar task [9]. The habenula ROI was created for each subject by manually identifying the left and right habenula based on the anatomical landmarks described by [19, 39]. As the VTA is involved in both reward and punishment learning, we included a probabilistic mask created by manual tracing for a prior study [40]. ROI placement is shown in Fig S2 and additional information on ROIs creation can be found in the Supplement. Parameter estimates of RPE and PPE contrasts were extracted from these ROIs and repeated measures ANOVAs were run in SPSS. In total, five ROIs were investigated; to protect again false positive results, a Bonferroni correction (*p* = 0.05/5 = 0.01) was used. A positive RPE beta identifies a brain region with higher activation for unexpected reward and lower activation for unexpected omission of rewards during gain condition (trials); conversely, a positive PPE beta identifies a brain region with higher activation for unexpected punishment and lower activation for unexpected omission of punishment during loss condition (trials).

#### PPI connectivity

Since animal studies have highlighted VTA-striatum and VTA-habenula pathways during reward and punishment learning, respectively, generalized PPI [41] was conducted using the VTA as the seed region. Using an ROI approach, parameter estimates (i.e., mean connectivity values) from the habenula and right striatum (see findings) were extracted for each condition (for completeness, the right insula and left striatum were also included). *Group* × *Valence* ANOVAs were run for each ROI.

## Results

Compared to controls, MDD individuals reported higher BDI-II, HDRS, and SHPS scores (Table 1). Groups did not differ in age, gender, socioeconomic status, ethnicity, and years of education.

**Table 1:** Demographics of participants in the final sample. Mean with standard deviations are reported

	Controls	MDD	*p* value
Sample size	26	25	N/A
Age	26.31 ± 7.96	25.25 ± 5.46	>0.5
Gender	19f, 7m	19f, 6m	>0.5
Caucasian	17 (65.4%)	16 (64%)	>0.5
Years of education	15.44 ± 1.80	15.80 ± 2.31	>0.5
BDI	0.44 ± 0.71	26.26 ± 9.21	<0.001
SHAPS (Anhedonia)	18.6 ± 4.49	33.40 ± 4.22	<0.001
HDRS	0.42 ± 0.94	17.27 ± 3.99	<0.001
Number of lifetime MDEs	N/A	3.72 ± 3.06	N/A
Age of initial onset	N/A	17.63 ± 5.34	N/A
Length of current episode (in months)	N/A	11.52 ± 15.41	N/A

*Note*: BDI-II: Beck Depression Inventory-II [36], SHAPS: Snaith Hamilton Pleasure Scale [37]; HDRS: Hamilton Depression Rating Scale (17-items; [35]). Missing data: 2 controls for race, 1 control for BDI and Anhedonia; 3 MDD for length of current episode and HDRS; 1 MDD for age of initial onset; 7 MDD for number of episodes.

### Task performance

**Fig. 1:**
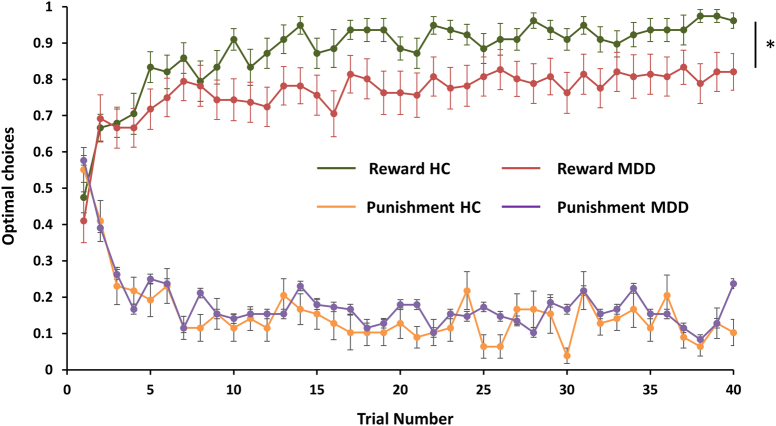
Behavioral results: Observed behavioral choices for reward [green—healthy controls; Red—MDD] and punishments [orange—healthy controls; purple—MDD] in controls vs. MDD. The learning curves depict, trial-by-trial, averaged proportion of “correct” stimulus (associated with a probability of 0.8 of winning money) in the gain condition (upper graph), and the “incorrect” stimulus (associated with a probability of 0.8 of losing money) in the loss condition (lower graph) chosen by participants. Error bars represent standard error.

Participants who selected less than 50% of correct stimuli (averaged across 3 runs) were excluded, as this might reflect task non-compliance. Based on this criterion, three MDD participants were excluded, leaving 26 controls and 25 MDD individuals for the analyses. A mixed-effects linear regression of choices (correct/incorrect) over trials revealed a *Group* × *Valence* interaction (*b* = −0.057, *p* = 0.022), driven by group differences during reward (*b* = −0.002, *p* = 0.002) but not punishment learning (*b* = −0.0003, *p* = 0.60). Specifically, relative to controls, the MDD group was characterized by reduced learning from rewards (i.e., fewer choices of the stimulus indicating a high probability of monetary gain), but no impairment in avoiding the stimulus associated with a high probability of monetary punishment (Fig. 1). Overall choice accuracy is listed in Table 2.

**Table 2:** Summary of task performance (choice accuracy as a function of trial type) in the healthy control (*N* = 26) and MDD (*N* = 25) groups. Mean with standard deviations are reported.

Variable	Reward		Punishment		Neutral	
	Controls	MDD	Controls	MDD	Controls	MDD
Choice accuracy (%)	87.82 ± 12.51	80.37 ± 19.18	83.11 ± 10.11	83.70 ± 8.46	49.39 ± 20.68	54.20 ± 18.53
Number correct (%)	71.06 ± 7.75	66.73 ± 12.07	67.31 ± 6.61	68.23 ± 5.01	49.84 ± 11.71	52.57 ± 10.43

### Computational model

Quantification of model fits during reward and punishment conditions indicated a good fit, with no differences between groups (Table S1).

For the fMRI analyses, a fixed alpha was chosen, and a learning model was fitted with a single set of parameters [42]. Specifically, we used averaged estimates of alpha calculated across all subjects during reward (alpha: 0.3) and punishment conditions (alpha: 0.4) separately. However, several control analyses were conducted to test potential confounds resulting from differences in learning rates (see Supplementary Section *Influence of Learning Rates on Model-Based fMRI*).

### Whole-brain analyses

Replicating prior reports [6, 7], controls exhibited RPE signals in the right putamen/NAc, insula, visual cortex. Among the MDD group, whole-brain analyses revealed RPE signals only in the insula and visual cortex. In contrasts, both controls and MDD exhibited PPE signals in the insula, midcingulate, habenula/thalamus, and midbrain. However, a whole-brain flexible factorial ANOVA did not reveal any main effects of *Group*, *Valence* or *Group* ×* Valence* interaction (Figs S3A and S3B; Figs S4A and S4B; Tables S2A and S2B). All clusters were *p* < 0.05 FWE cluster-corrected.

### ROI analyses

Two ANOVAs for RPE and PPE ROIs were run individually.

#### Striatal ROI

**Fig. 2:**
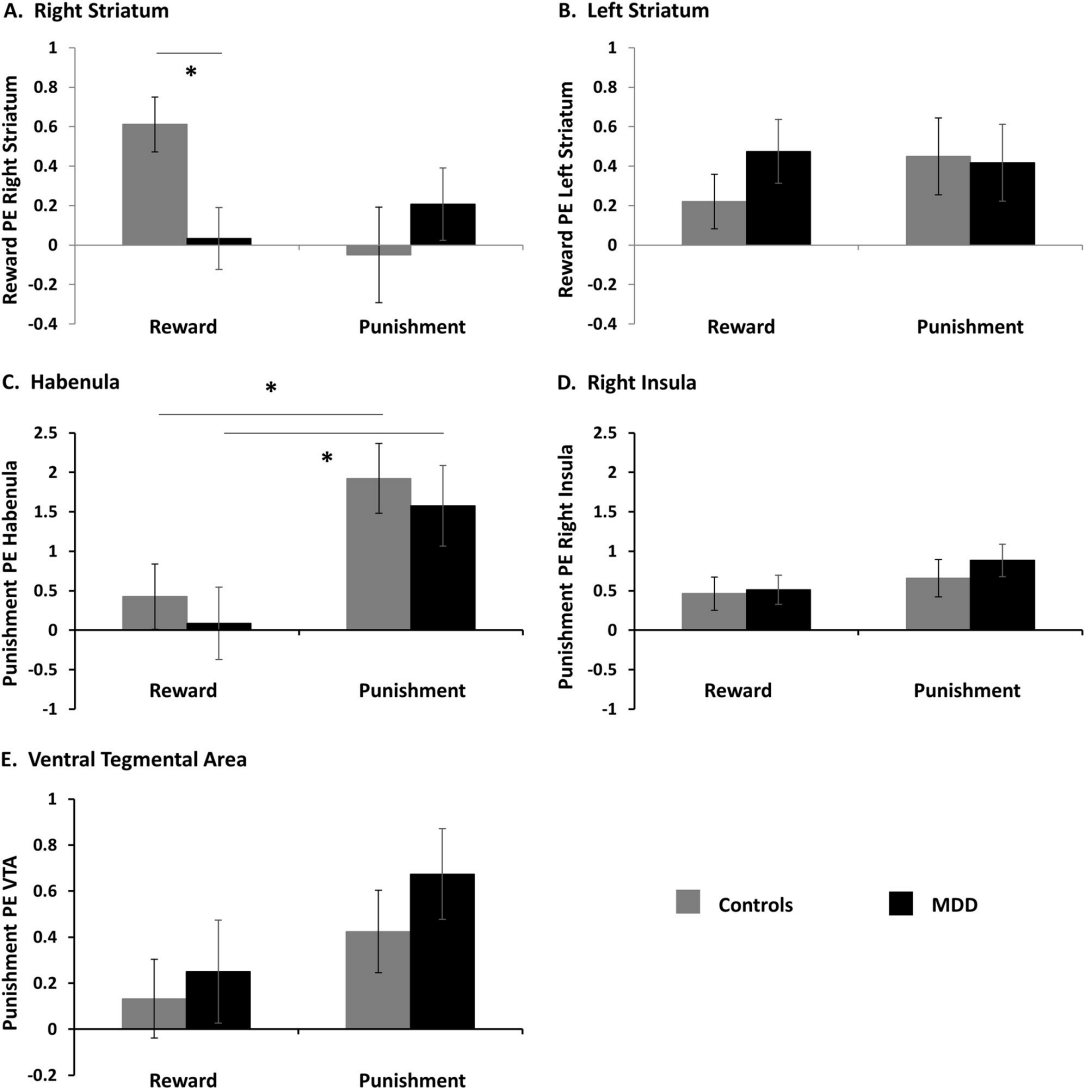
ROI results: Parameter estimates reflecting reward and punishment prediction errors extracted from the right (**a**) and left (**b**) striatum, habenula (**c**), right insula (**d**) and ventral tegmental area (VTA) (**e**) in healthy controls and MDD. Error bars represent standard error. *p < 0.05

A significant 3-way *Group* × *Valence* × *Hemisphere* (Left and Right striatum) interaction emerged [*F*(1,49) = 12.46, *p* = 0.001, η^2^*p* = 0.20). Follow-up analyses revealed a *Group* × *Valence* interaction for the right [*F*(1,49) = 4.52, *p* = 0.04, η^2^*p* = 0.08], but not left [*F*(1,49) = 0.76, *p* = 0.39, η^2^*p* = 0.02] striatum (Fig. 2a,b). To formally test for laterality effects, we conducted a *Group* × *Hemisphere* ANOVA for each *Valence* separately; for RPE, there was a significant *Group* × *Hemisphere* interaction [*F*(1,49) = 14.91, *p* < 0.001], whereas the interaction was not significant for PPE [*F*(1,49) = 1.3, *p* = 0.26]. Post-hoc analyses further revealed that the right striatum finding was driven by blunted RPE [*t*(49) = 2.77, *p* = 0.008, ds = 0.77], but similar PPE signaling [*t*(49) = −0.84, *p* = 0.40, ds = 0.24] in the MDD group, relative to controls. In addition, similar RPE [*t*(49) = −1.20, *p* = 0.24, ds = 0.33] and PPE signaling [*t*(49) = 0.12, *p* = 0.9, ds = 0.03] were observed in the left striatum across both groups. The group difference in the right striatum survived after applying correction for multiple comparisons (Bonferroni correction *p* = 0.01).

#### Habenula, VTA and insula ROIs

A *Group* × *Valence* ×* ROI* (Habenula, VTA, Insula) ANOVA revealed a significant main effect of *Valence* [*F*(1,49) = 12.52, *p* = 0.001, η^2^*p* = 0.20], a main effect of *ROI* [*F*(1,49) = 5.21, *p* = 0.007, η^2^*p* = 0.096] and a *Valence* × *ROI* interaction [*F*(1,49) = 5.15, *p* = 0.007, η^2^*p* = 0.095), but these effects did not interact with *Group*. Follow-up tests showed larger PPE than RPE in the habenula [*t*(50) = −3.27, *p* = 0.002; Fig. 2c] and a trend in the VTA [*t*(50) = −1.69, *p* = 0.09; Fig. 2e] across participants, but no difference in the insula [*t*(50) = −1.39, *p* = 0.17; Fig. 2d].

### Correlation with clinical variables

Unlike prior studies [21, 22, 26], RPE signal in the right striatum did not correlate with anhedonia scores within the MDD group. However, right striatal RPE correlated with depressive symptoms as measured by BDI (*r* = −0.43; *p* = 0.032; Fig S5).

**Fig. 3:**
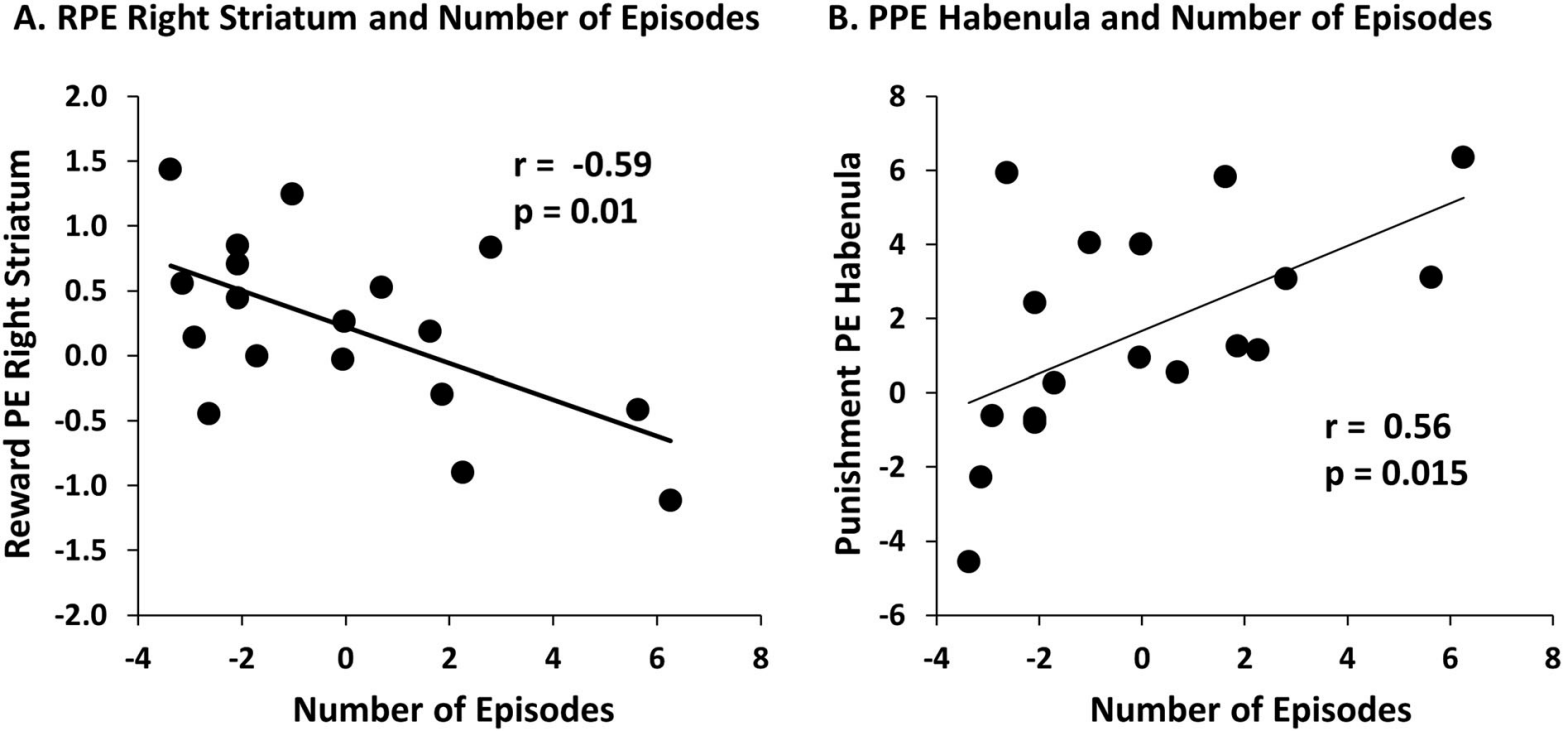
Correlation between number of depressive episodes, and (**a)** reward prediction error in the right striatum and (**b**) punishment prediction error in the habenula, in the MDD group. [*Note*: To evaluate the effect of number of MDEs on PE without potential confounds, these analyses were conducted while adjusting for length of current episode. Unstandardized residuals are shown in the figure; correlation plots with raw scores (with no covariates) are shown in the Supplementary Fig S6]. These correlations were significant even after controlling for both length of current episode and current depression severity (BDI scores) [right striatum: *r* = −0.60; *p* = 0.011 and habenula: *r* = 0.56; *p* = 0.018]. Information about number of episodes was missing for seven MDD individuals, so the sample size for this correlational analysis was *N* = 18. PE - prediction error.

In addition, number of major depressive episodes (MDEs) (controlled for length of current episode; *n* = 18) correlated negatively with RPE signals in the right striatum (*r* = −0.59, *p* = 0.010; Fig. 3a), but positively with PPE signals in the habenula (*r* = 0.56 *p* = 0.015; Fig. 3b). That is, an increasing number of MDE was associated with more blunted reward signals in the right striatum but enhanced punishment signals in the habenula. These associations survived even after controlling for both length of current episode and current depression severity (BDI scores) [right striatum: *r* = −0.60; *p* = 0.011 and habenula: *r* = 0.56; *p* = 0.018], highlighting an effect of disease burden. These correlations were also confirmed when considering number of episodes (without any covariates) and right striatal RPE (*r* = −0.54, *p* = 0.020) and habenula PPE (*r* = 0.56, *p* = 0.016) (see Fig S6 for scatterplots with raw scores). However, they did not survive a Bonferroni correction for the ten correlations that were performed [*p* = 0.05/10 = 0.005; (age of onset and numbers of MDEs) × (right and left striatum, habenula and VTA) + (anhedonia and BDI) × right striatum; *n* = 10].

### PPI connectivity

**Fig. 4:**
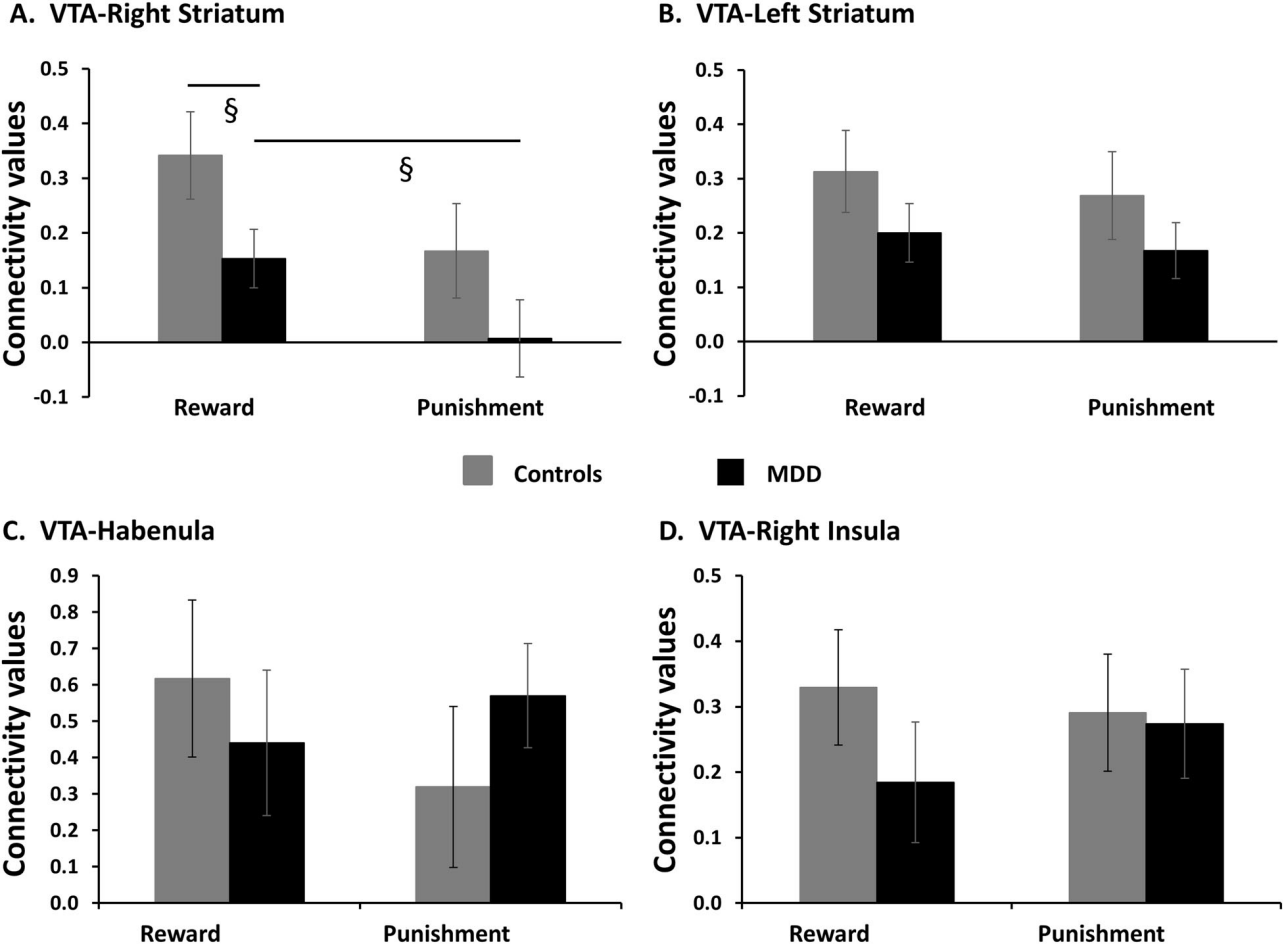
VTA-Right Striatum (**a**), VTA-Left Striatum (**b**), VTA-Habenula (**c**), and VTA-Right Insula (**d**) connectivity values in healthy controls and MDD. Error bars represent standard error. ^§^*p* < 0.1

A *Group* × *Valence* ANOVA of VTA-right striatum connectivity revealed main effects of *Valence* [*F*(1,49) = 4.74, *p* = 0.034, η^2^*p* = 0.09] and *Group* [*F*(1,49) = 5.34, *p* = 0.025, η^2^*p* = 0.10; Fig. 4a]. Overall, participants had a greater VTA-right striatum connectivity during gain vs. loss trials and MDD individuals had an overall reduction in VTA-right striatum connectivity when compared to controls. Moreover, exploratory analyses revealed RPE in the VTA correlated positively with RPE in the right striatum across both groups [*r* = 0.36, *p* = 0.009], mainly driven by controls [*r* = 0.57, *p* = 0.002] and not MDD [*r* = 0.29, *p* = 0.15], but these correlations did not significantly differ [*z* = −1.14, *p* = 0.3; Fig S7]. The VTA-left striatum, VTA-habenula, and VTA-Insula (Fig. 4b–d) connectivity were not different between groups or valence.

## Discussion

Using a monetary instrumental learning task, we investigated neural mechanisms underlying reward and punishment learning in psychiatrically healthy and unmedicated MDD individuals. Two central findings emerged. First, relative to controls, MDD participants were characterized by reduced learning from monetary rewards, but no impairment in avoiding the stimulus associated with a high probability of monetary punishments. Neurally, the MDD group showed blunted RPE signaling in the right (but not left) striatum as well as overall impaired connectivity between the VTA and right striatum during feedback. Highlighting the specificity of these findings, groups did not differ in regions encoding PPE signals. Second, within the MDD group, number of MDEs was associated with weaker RPE in the right striatum, and enhanced PPE in the habenula. Collectively, these findings highlight behavioral and neural evidence of disrupted incentive learning in unmedicated MDD, with abnormalities increasingly pronounced with disease burden.

DA neurons in the VTA have been hypothesized to generate RPE signals that are then transmitted to the striatum and cortex for value computations and action selection. Few studies have investigated the VTA-striatal pathway during learning in humans [30, 32, 33]. Using PPI, we found stronger functional connectivity between the VTA and right striatum during gain vs. loss trials across both groups. MDD individuals exhibited overall reduced connectivity between these regions during feedback. In addition, RPE signals in the right striatum positively correlated with RPE signals in the VTA, but significantly only among controls. Collectively, these results highlight that, in contrast to controls, the MDD group failed to show robust functional connectivity between these regions during reward learning. This raises the possibility that, in MDD, RPE signals are not appropriately transmitted to the striatum due to reduced connectivity between these two regions, thereby causing reduced downstream RPE signaling and impaired reward learning. Supporting this interpretation, a recent study reported reduced functional connectivity between the VTA, striatum and prefrontal cortex in MDD individuals unresponsive to repetitive transcranial magnetic stimulation of the dorsal medial prefrontal cortex; these individuals also exhibited higher baseline anhedonia when compared to responders [31]. More recently, Rutledge and colleagues observed intact striatal RPE signals in the MDD group during a non-learning task, suggesting that the computation of a DA RPE signal is intact in MDD [23]. These authors interpreted prior observations of blunted striatal RPE during learning tasks to be due to a downstream DA signaling deficit, rather than a fundamental failure of the DAergic encoding of RPEs. This is critical for the interpretation of our findings, as only in controls, the VTA RPE correlated with striatal RPE. However, the weakened VTA-striatal connectivity in the MDD group points to a downstream DA signaling deficit, that then leads to impaired reward learning [23].

Consistent with prior studies in healthy controls [17–19], we observed PPE signals in a habenula/thalamus cluster in both groups. The extensive influence of habenula neurons on the dopaminergic pathway highlights this region’s critical role in processing motivationally salient stimuli [15]. Both hyperactive and hypoactive habenular activation during punishment processing have been reported in depressive behaviors. For instance, prior animal studies have reported elevated habenula metabolism [43] and enhanced excitatory inputs to VTA-projecting habenula neurons during learned helplessness [44], with the former reversed following antidepressant treatment [45]. In contrast, Lawson and colleagues [28] reported that unmedicated MDD subjects exhibited reduced negative task-related (phasic) habenula responses only during primary aversive conditioning, but not during monetary loss. In our study, we found no group differences in habenula activation or VTA-habenula connectivity during either gain or loss conditions. However, PPE signals in the habenula correlated positively with number of depressive episodes, suggesting that punishment-related habenula activation increases with disease burden, in partial support of habenula hyperactivity during punishment processing reported in prior literature in MDD individuals [46, 47].

In addition to the VTA and habenula, PPE signals were observed in the midcingulate, midbrain (periaqueductal gray), insula and thalamus across both groups, consistent with other studies [6, 48]. Contrary to our hypotheses, we observed both RPE and PPE signals in the insula. Even though the insula is consistently activated during aversive conditioning with different types of stimuli (e.g., shock, monetary loss, social rejection), it also emerges during encoding of RPE [49, 50], suggesting that the insula might encode a salience PE [51, 52].

There are four limitations that warrant mention. First, even though we replicated prior finding of blunted RPE signal in the striatum, this was specific to the right striatum; these laterality effects warrant independent confirmations, because they were not hypothesized a priori. Second, although the sample size was comparable or larger than prior studies in this area [21–23], replication with bigger samples will be important. Third, our correlational findings, although interesting, did not survive Bonferroni correction for multiple comparisons, hence, these results await replications. Lastly, despite careful quality control checks during registration, results from the habenula ROI should be interpreted with caution, as fMRI resolution is limited for small structures.

In summary, we found that MDD individuals were characterized by reduced VTA-striatum connectivity during feedback and blunted downstream RPE signaling in the striatum, and overall impaired reward learning. Highlighting the specificity of these findings, the groups did not differ in punishment learning and individuals with MDD encoded PPE signals in the insula, VTA, midcingulate and habenula as well as the controls did. However, number of depressive episodes modulated RPE and PPE signals, suggesting the importance of disease burden on learning. Collectively, these findings highlight important reward-related learning deficits in MDD and their underlying pathophysiology.

## Electronic supplementary material

Supplementary Methods(PDF 214 kb)

Supplementary Figures(PDF 2498 kb)
