# Supplementary material for: Impaired reward prediction error encoding and striatal-midbrain connectivity in depression
Source: Neuropsychopharmacology. 2018 Feb 26;43(7):1581–8. doi: 10.1038/s41386-018-0032-x (PMC5983542; doi:10.1038/s41386-018-0032-x)
Supplement: Supplementary file 1 — Supplementary Methods(PDF 214 kb) [file 41386_2018_32_MOESM1_ESM.pdf]

Impaired reward prediction error encoding and striatal-midbrain connectivity in depression

Kumar P, Goer F, Murray L, Dillon DG, Beltzer ML, Cohen AL, Brooks NH, Pizzagalli, DA

Supplementary Material

METHODS

Participants

Twenty-six healthy controls (mean age: 26.31±7.96; 19 females) and 28 unmedicated individuals with MDD (mean age: 25.50±5.42; 22 females) participated in this study. All participants provided written informed consent to a protocol approved by the Partners Healthcare and McLean Hospital IRB. Participants were right-handed and reported no medical or neurological illnesses, no contraindications to MRI, no lifetime substance dependence and no substance abuse in the past year. All individuals were assessed using the Structured Clinical Interview for the DSM-IV [SCID (First *et al*, 2002)] to confirm study eligibility and MDD participants had to have a diagnosis of major depressive disorder according to the SCID. In addition, information about the number of prior major depressive episodes (MDE), length of current MDE and age of onset of the first MDE were collected. All participants were also assessed using the 17-item Hamilton Depression Inventory (Hamilton, 1980). Exclusion criteria for the depressed group included use of any psychotropic medication in the past 2 weeks (6 weeks for fluoxetine, 6 months for dopaminergic drugs or antipsychotics) and a psychiatric history of other major axis I disorders (except social and generalized anxiety if they were secondary to MDD). Exclusion criteria for the control group included current or history of psychiatric illnesses (assessed by SCID) and a family history of mood disorders or psychosis.

Three MDD participants had a current comorbidity of social phobia (secondary to MDD) and four MDD participants had a past diagnosis of social phobia. Data from the 3rd run of two participants (one HC due to excessive movement and one MDD due to scanner technical difficulties) were excluded from the analyses; for these participants, data from first two runs were averaged.

Instrumental Reinforcement Learning (RL) Task

After a short practice outside the scanner, participants performed three runs of an instrumental learning task [adapted from (Pessiglione *et al*, 2006)] with monetary outcomes, each

time with new pairs of stimuli (letters from the Agathodaimon font; Fig S1). Briefly, during each run of 120 trials (40 gain, 40 loss and 40 neutral), participants were presented with one of three pairs of stimuli (gain, loss and neutral), which were associated with 80%/20% probabilities of the following: Gain ($10/Nothing), Loss (Nothing/-$10), Neutral ($0/Nothing). On each trial, the stimuli from one of the pairs were presented side by side (position counterbalanced across trials) and participants were asked to choose one of them. A red arrow was presented below the chosen stimulus for a total cue presentation time of 2.5s. After a jittered inter-stimulus interval (ISI), feedback was presented for 1.5s. The next trial started after a fixed inter trial interval (ITI) of 0.5s. To win money, participants had to learn, by trial and error, the stimulus-outcome contingency. Each run lasted ~7 min and included new pairs of stimuli. Participants were told that one of the runs would be randomly selected for the total winnings (in reality, they were all given the same fixed amount of $50). Task accuracy during gain, loss and neutral trials are summarized in Table 1 (main text).

Computational Model (Q-Learning)

A standard Q-learning algorithm was used to calculate the expected value of choices and prediction error based on individual's choice and feedback history (Sutton and Barto, 1998). For each trial, the model estimated the expected value of A (Q_A) and B (Q_B), which corresponds to the expected reward obtained by choosing a cue. Q values were set to zero at the beginning of each run. After every trial, $Q_A(t)$ or $Q_B(t)$ were updated based on the feedback participants received in that R(t) trial, per the following rule:

$$Q_{choosen\_cue}(t+1) = Q_{choosen\_cue}(t) + \alpha\, \delta(t),$$

where α is the learning parameter and δ is the prediction error. Central to learning is the prediction error ($\delta(t)$), which is defined as the deviation between expected feedback [Qchoosen_cue (t)] and the actual feedback [R(t)] or

$$\delta(t) = R(t) - Q_{choosen\_cue}(t),$$

where R is assigned 1 and 0 for reward and no reward outcomes, respectively, during gain trials, and assigned 0 and -1 for no punishment and punishment outcomes, respectively, during loss trials. Based on the Q values at any given trial, the probability of choosing a particular stimulus was calculated using the softmax rule, such as the probability of choosing stimulus A was

$$P_A(t) = \exp(Q_A(t)/\beta)/[\exp(Q_A(t)/\beta) + \exp(Q_B(t)/\beta)]$$

The two free parameters, alpha (α; learning rate) and beta (β; temperature) were optimized for every subject to maximize the probability of actual choices under the model using maximum likelihood estimation and fmincon function in MATLAB. Specifically, for each participant, the free parameters' space was searched to identify parameters that would maximize the likelihood of their own trial-by-trial sequence of choices, using multiple random starting points. The learning parameter (α) represents the influence of the feedback on the subjective values of the chosen stimulus and varies between 0 and 1. For example, a low α reflects a relatively small impact of the prior feedback on the current decision, whereas a higher α indicates a larger impact of feedback. The temperature parameter (β) specifies noise that reflects the accuracy of response choice (Sutton and Barto, 1998). For example, the β estimate is high if participants randomly choose between the two stimuli, and these choices do not correlate with the subjective value of the two stimuli. Conversely, if participants always choose the stimulus with the higher subjective value, then β is close to zero (which is mostly in this task). Average learning rate and beta values are listed in Table S1. Overall, participants had a smaller alpha and beta for reward than punishment condition ($p < 0.05$; Table S1). However, no group differences were observed in either condition ($p > 0.5$).

Comparison of Q-learning Model Fits Between Groups

We tested how well the reward learning model fitted the observed data compared with chance by estimating a naïve model assuming that participants choose all stimuli with equal probability and had no free parameters. To do this, we calculated pseudoR^2 values defined by pseudo$R^2 = 1 - (LLE_{model}/LLE_{chance})$. LLE_{model} corresponds to the maximum logarithmic likelihood of the observed choices under the model. LLE_{chance} corresponds to the logarithmic likelihood of choices at chance [$LLE_{chance} = t*\log(0.5)$], t being the number of trials. To test if there are potential differences in model fit between groups, two sample independent t-tests were run for reward and punishment conditions separately. Analysis of pseudoR^2 revealed no differences between healthy controls and MDD groups in both the gain (controls: 0.77±0.20; MDD: 0.68±0.25; $t(49)=1.43$; $p=0.16$) and punishment (controls: 0.43±0.20; MDD: 0.48±0.18; $t(49)=-0.98$; $p=0.33$) condition. Overall, our model performed better in the gain than loss condition ($p<0.001$; Table S1). There was only one participant whose Q-learning model fit was poorer than the null model. When we repeated the analyses with this person excluded, the results did not change.

Functional Imaging and Analyses

fMRI Data Acquisition

A 3T Tim Trio Siemens scanner (Siemens Medical Systems, Iselin, N.J.) equipped with a 32-channel head coil was used to acquire the MRI data. High-resolution structural data were acquired using a T1-weighted magnetization-prepared rapid acquisition with gradient multi echo (MPRAGE) imaging sequence with the following acquisition parameters: repetition time (TR) = 2200 ms; echo times (TE) = 1.54, 3.36, 5.18 and 7 ms; field of view = 230 mm; voxel dimensions = 1.2 x 1.2 x 1.2 mm^3; 144 slices. Functional MRI data were acquired using a gradient echo T2*-weighted echo planar imaging sequence with 30 degree titled slice acquisition to recover signal in regions affected by susceptibility artifacts (Deichmann *et al*, 2003) with the following acquisition parameters: repetition time (TR) = 3000 ms; echo time (TE) = 30 ms; field of view = 224 mm; voxel dimension = 3.5 x 3.5 x 2.0 mm; 57 interleaved slices and a GRAPPA acceleration factor of 2.

fMRI Data Pre-processing

Functional MRI data were preprocessed and analyzed using Statistical Parametric Mapping software (SPM12; http://www.fil.ion.ucl.ac.uk/spm). After initial quality control of hardware-related artifacts in the raw images, distortion correction was applied using field maps acquired in the task session. Functional images were then realigned to the mean image of the series, corrected for motion and slice timing related artifacts, co-registered with the anatomical image, normalized to the 2x2x2 mm MNI template, and smoothed with a 4mm Gaussian kernel

Statistical Analyses

First-level general linear model included six regressors (cue and outcome presentation onsets during reward, punishment and neutral trials). In addition, outcome onset times for rewards and punishments were parametrically modulated by model-derived reward and punishment prediction error, respectively, and convolved with a hemodynamic response function. The covariates of no interest included the cue and outcome during neutral trials, six motion realignment parameters, and a constant term modelling the baseline of unchanged neural activity.

ROI Analyses

A staged ROI selection was implemented. Specifically, priority was given to clusters emerging from meta-analyses probing PE; for small regions hypothesized to be implicated in PE (VTA, habenula), clusters based on manual identification were used to avoid potential biases.

Finally, for larger and functionally heterogenous regions (e.g., insula), a sphere was drawn around the coordinates emerging from prior studies using the same paradigm as used here. Based on these considerations, anatomically constrained bilateral striatum were extracted from a recent meta-analysis of RPE studies in healthy controls (Chase *et al*, 2015). These anatomical masks were created from the FSL Harvard-Oxford subcortical atlas using 40% probability threshold. As prior studies have reported PPE signals in the right insula (Pessiglione *et al*, 2006) and habenula (Hennigan *et al*, 2015; Lawson *et al*, 2014; Salas *et al*, 2010), a right insula mask was created by drawing a sphere with 10mm diameter around the peak voxel (40, 28, -6) reported in (Pessiglione *et al*, 2006). The habenula ROI was created for each subject by manually identifying one voxel centered each in the left and right habenula in the normalized T1 of every subject based on the anatomical landmarks described by (Lawson *et al*, 2013; Salas *et al*, 2010). Since the habenula is a small structure of approximately ~30mm^3, which corresponds to one 3x3x3 voxel, we extracted beta weights only from this voxel identified in the structural image. Due to the extensive white matter plexuses contained within the habenula, this structure's density appears brighter than the adjacent thalamic grey matter on T1-weighted images, aiding its delineation from surrounding grey matter and cerebrospinal fluid [CSF (Salas *et al*, 2010)]. The beta weights from the left and right habenula voxel were averaged. Additionally, since the VTA is involved in both reward and punishment learning, we included a probabilistic mask created by manual tracing (Murty *et al*, 2014) thresholded by 60% to ensure accurate anatomical constrains. ROI placement is shown in supplementary Fig S2. All ROIs were multiplied by a group mask created across all subjects, to ensure all subjects had identical voxels within these ROIs. Parameter estimates of RPE and PPE contrasts were extracted from these five ROIs and mixed ANOVAs were run in SPSS. In total, five ROIs were investigated; to protect again false positive results, a Bonferroni correction (p=0.05/5=0.01) was used. A positive RPE beta identifies a brain region with higher activation for unexpected reward and lower activation for unexpected omission of rewards during gain condition (trials); conversely, a positive PPE beta identifies a brain region with higher activation for unexpected punishment and lower activation for unexpected omission of punishment during loss condition (trials).

Whole-brain analyses

See Main text (and Figs S3A & S3B; Figs S4A & S4B; Tables S2A & S2B).

Correlations with Behavioral and Clinical Variables

No correlations were observed between reward accuracy and RPE signal in the right striatum (p > 0.5). However, habenula PPE signal correlated with punishment accuracy (*r*=0.29, *p*=0.042) across all subjects. The VTA-right striatal and VTA-habenula connectivity strengths did not correlate with reward and punishment accuracy, respectively, or number of depressive episodes (*p*>0.5).

Unlike prior studies (Chase *et al*, 2015; Gradin *et al*, 2011; Kumar *et al*, 2008), RPE signal in the right striatum did not correlate with anhedonia scores within the MDD group. However, right striatal RPE correlated with depressive symptoms as measured by BDI (*r*=-0.43; *p*=0.032; Fig S5). Within the MDD group, age of first MDE correlated positively with RPE signals in the right striatum (*r*=0.41, *p*=0.046) and negatively with PPE in the habenula (*r*=-0.46 *p*=0.024). However, these correlations became non-significant when controlling for current age and time since first episode (RPE right striatum: *r*=0.24, *p*=0.25; PPE habenula: *r*=-0.33 *p*=0.11). Conversely, and as described in the main text, number of prior depressive episodes correlated negatively with RPE signals in the right striatum (*r*=-0.59, *p*=0.010), but positively with PPE signals in the habenula (*r*=0.56, *p*=0.015; Fig 3, main text). Since these associations remained when controlling for length of current episode and current depression severity (BDI scores), overall disease burden appeared to drive these effects. Finally, and highlighting the robustness of these findings, the correlations were confirmed when considering raw number of episodes (without any covariates) and right striatal RPE (*r*=-0.54, *p*=0.020; Fig S6A) and habenula PPE (*r*=0.56, *p*=0.016; Fig S6B).

PPI Connectivity Analyses

We investigated functional connectivity between VTA-habenula-striatum during reward and punishment trials using the generalized PPI toolbox (Friston *et al*, 1997; McLaren *et al*, 2012), The VTA was used as the seed and time-series were extracted from individual subjects. For each subject, subject-level GLMs were constructed as described above with the addition of the VTA seed time-series and two additional PPI regressors that are the respective product of the seed time-series and the regressors for reward and punishment. These regressors are orthogonal to the task and seed regressors, and thus describe the contribution of the interaction above and beyond the main effects of the task and seed time-series (McLaren *et al*, 2012). The parameter estimates (connectivity value) of the two PPI regressors reflect the correlation between activity in the VTA

and activity in every other voxel during reward and punishment trials, respectively. Since we had a priori hypotheses, we extracted the mean connectivity values from the habenula, insula and right and left striatum. Exploratory analyses revealed RPE in the VTA correlated positively with RPE in the right striatum across both groups [r=0.36, p=0.009], mainly driven by controls [r=0.57, p=0.002] and not MDD [r=0.29, p=0.15], but these correlations did not significantly differ [z=-1.14, p=0.3; Fig S7].

Influence of Learning Rates on Model-Based fMRI

Usually, there are three main strategies for determining the learning rate: fixed, group-fixed and individual learning. Whereas individual learning is better at accommodating subjects' behaviors (Estes and Maddox, 2005), fixed learning reduces noise and may provide a form of regularization, improving reliability at the expense of losing individual learning rate data (Chase *et al*, 2015; Chen *et al*, 2015; Daw, 2011). Moreover, a recent report suggested that mis-specifying learning rates in tasks with a fixed reward distribution does not affect model-based fMRI fit (Wilson and Niv, 2015). To evaluate this possibility in the current sample, we calculated PEs from our model with learning rates varying from 0.01 to 0.99, in steps of 0.01 and conducted fMRI analyses using different learning rates. Correlation between the beta weights as a function of learning rates for each ROI were calculated and plotted.

We found strong correlations (Pearson r > ~0.80) between beta weights extracted from models estimated with learning rates varying from 0.01 to 0.99 in increments of 0.01. Correlation maps for the right striatum, right insula and VTA are shown in Fig S8-S10 (the left striatum and habenula showed similar patterns, and findings are available upon request). In addition, Fig S11 shows the line plots for the right striatum, where each line represents beta weights extracted from models using different learning rates across subjects. Although the plots for each subject indicated that fMRI estimates are not affected by learning rates, the curves for individual subjects do show that there might be a maximum beta that is optimal for each subject. To evaluate this possibility, we re-ran fMRI analyses using individual learning rates for each subject. Overall, similar spatial maps emerged when using individual learning rates (although the cluster size was smaller with individual learning; see Figs S12A & S12B and Supplementary Tables 3A, 3B for voxel coordinates). Most importantly, functional group differences (as shown by mean beta and effect sizes, Fig S13) remained unchanged. Effect sizes were similar across our ROIs (Fig S13).

Collectively, these control analyses indicate that differences in learning rates did not influence the fMRI results. Specifically, we did not find any differences in group results either using group-fixed or individual learning rate for estimating prediction error signals. In addition, we observed a strong correlation between beta weights extracted from different learning rate models across subjects, replicating the findings by (Wilson and Niv, 2015). Results from our study suggest that many analyses will be valid even if the parameters cannot be well estimated from behavior (Wilson and Niv, 2015).

FIGURE LEGENDS

Supplemental Figure 1: Design of the monetarily reinforced instrumental learning task. Participants selected either the left or right of two visual stimuli presented on the screen. Their selection was shown by a red arrow under the chosen stimuli for the remainder of 2.5s. Following a jittered interstimulus interval (ISI), outcome depending on the type of the trial was shown for 1.5s. The next trial then began after a fixed inter-trial interval of 0.5s. In the gain trial (top row), the chosen stimulus was associated with a probability of 0.8 of winning $10 and a probability of 0.2 of winning nothing. Similarly, in the loss trial (middle row), the chosen stimulus was associated with a probability of 0.8 of losing $10 and a probability of 0.2 of losing nothing. Neutral trials were associated with no change.

Supplemental Figure 2: Regions-of-Interest (ROI) placement. (A) Striatal ROIs from a prior meta-analysis of RPE studies (Chase et al, 2015). (B) Ventral tegmental area created by manual tracing [obtained from Adcock's Lab, Duke University], thresholded at 60%. (C) Habenula mask obtained from combining peak voxels identified from structural images of each subject. [Note: This analysis was done by extracting beta weights from individual voxels of each subject, this mask is only for visualization]. (D) Right Insula, created by drawing a 10mm sphere around the peak voxel (40, 28, -6) obtained from (Pessiglione et al, 2006).

Supplemental Figure 3: Whole-brain results: Brain activity correlating with reward prediction errors in controls (A) and MDD (B) derived from the computational model. Clusters are $p < 0.05$ family-wise error corrected, with an initial cluster forming threshold of $p < 0.005$.

Supplemental Figure 4: Whole-brain results: Brain activity correlating with punishment prediction errors in controls (A) and MDD (B) derived from the computational model. Clusters are $p < 0.05$ family-wise error corrected, with an initial cluster forming threshold of $p < 0.005$.

Supplemental Figure 5: Correlation between RPE signals in the right striatum and BDI within the MDD group.

Supplementary Figure 6: Correlation between number of depressive episodes (without any covariates) and (A) reward prediction error in the right striatum and punishment prediction error in the (B) habenula, in the MDD group. Information about number of episodes was missing for 7 MDD individuals, so the sample size for this correlational analysis was N=18. PE = prediction error.

Supplemental Figure 7: Correlation between RPE activation in the right striatum and VTA across both groups. Red – MDD; Green – Healthy controls.

Supplemental Figure 8: Correlation map showing Pearson r values between beta weights of RPE signals in right striatum in (A) Healthy controls and (B) MDD; correlation map between PPE signals in right striatum in (C) Healthy controls and (D) MDD extracted from different learning rates.

Supplemental Figure 9: Correlation map showing Pearson r values between beta weights of RPE signals in right insula in (A) Healthy controls and (B) MDD; correlation map between PPE signals in right insula in (C) Healthy controls and (D) MDD extracted from different learning rates.

Supplemental Figure 10: Correlation map showing Pearson r values between beta weights of RPE signals in VTA in (A) Healthy controls and (B) MDD; correlations between PPE signals in VTA in (C) Healthy controls and (D) MDD extracted from different learning rates. [Note: habenula and left striatum not shown, but identical results were obtained.]

Supplemental Figure 11: Line plots representing beta weights of RPE signals in the right striatum extracted from different learnings in controls (top) and MDD (bottom). Each line represents one subject. [Note: other ROIs showed similar results.]

Supplemental Figure 12: Whole-brain results using individual learning rates: Brain activity correlated with (A) reward prediction errors and (B) punishment prediction errors derived from the computational model in healthy controls. For ease of comparison and visualization, identical initial cluster forming threshold of $p = 0.005$ and extent threshold of 50 voxels were used as in the main analyses.

Supplemental Figure 13: Beta weights of (A) RPE and (B) PPE signals extracted from the right striatum, VTA, right insula and habenula from fixed and individual learning maps. Effect sizes were calculated using Cohen's d of (C) RPE and (D) PPE beta weights in these ROIs from fixed and individual learning maps. Error bars represent standard error.

Supplementary Table 1: Model parameters (reward and punishment trial type) in the Healthy Control (N=26) and MDD (N=25) group. Mean with standard deviations are listed.

Variable	Reward		Punishment	
	Controls	MDD	Controls	MDD
PseudoR^2	0.77±0.20	0.68±0.25	0.43±0.20	0.48±0.18
LLE Model	6.34±5.59	8.78±6.9	15.57±5.45	13.98±4.92
Learning Rate (α)	0.31±0.14	0.28±0.14	0.38±0.23	0.42±0.24
Temperature (β)	0.11±0.16	0.13±0.15	0.23±0.17	0.19±0.20

Supplementary Table 2: MNI peak coordinates of brain regions encoding Reward Prediction Error (Panel A) and Punishment Prediction Error (Panel B) in the Healthy Control (N = 26) and MDD (N = 25) group, modeled with fixed group average reward learning rate = 0.3, punishment learning rate = 0.4; $p < 0.05$ Family Wise Error (FWE) cluster corrected, with an initial cluster forming threshold of p = 0.005. NAc: nucleus accumbens

A. Reward Prediction Error

Brain Region	Cluster size	MNI (x, y, z)	Z score	Cluster p (FWE)
Healthy Controls				
Right Visual Cortex	7030	28, -78, -10	6.77	0.000
Right Calcarine Cortex	313	14, -68, 10	4.61	0.000
Left Superior Parietal Cortex	125	-30, -44, 40	4.54	0.006
Right Anterior Insula	144	34, 20, 4	3.91	0.002
Right Putamen/NAc	128	14, 14, -12	3.78	0.006
MDD				
Left Visual Cortex	9092	-26, -74, -12	6.74	0.000
Left Inferior Frontal Gyrus	172	-46, 6, 22	5.17	0.001
Left Superior Parietal Cortex	96	-32, -46, 54	4.55	0.040
Left Anterior Insula	100	-44, 18, -8	4.15	0.032
Posterior Cingulate	191	2, -34, 30	3.81	0.000
Right Precentral Gyrus	123	38, 2, 24	3.72	0.009

B. Punishment Prediction Error

Brain Region	Cluster size	MNI (x, y, z)	Z score	Cluster p (FWE)
Healthy Controls				
Right Visual Cortex	5706	34, -64, -14	6.18	0.000
Left Fusiform Gyrus	2763	-26, -66, -16	5.54	0.000
Thalamus/Habenula (part of fusiform gyrus cluster)		2, -30, -2	4.74	0.000
Mid Cingulate	1640	-2, 14, 46	5.02	0.000
Left Anterior Insula	828	-38, 14, 0	4.99	0.000
Right Middle Frontal Gyrus	113	42, 8, 40	4.58	0.020
Right Middle Frontal Gyrus	151	46, 24, 20	3.68	0.003
Right Insula	147	44, 12, 0	4.44	0.004
Precuneus	102	-4, -64, 50	4.09	0.037
Left Precentral Gyrus	264	-40, 4, 28	3.74	0.000
MDD				
Right Visual Cortex	4263	30, -86, 14	6.77	0.000
Midbrain/Thalamus/Habenula (part of visual cortex cluster)		6, -24, 2	4.14	0.000
Left Fusiform Gyrus	4825	-40, -78, -12	5.75	0.000
Mid Cingulate	1363	-6, 16, 50	5.41	0.000
Right Calcarine Cortex	388	18, -66, 12	5.26	0.000
Left Anterior Insula	408	-32, 26, 0	5.19	0.000
Right Inferior Frontal Gyrus	391	32, 28, -6	5.13	0.000
Right Middle Frontal Gyrus	379	44, 14, 30	4.90	0.000
Left Middle Frontal Gyrus	107	-36, 24, 22	4.36	0.026
Left Precentral Gyrus	528	-42, 8, 28	4.72	0.000

Right Supramarginal Gyrus	131	52, -30, 50	4.02	0.007
Rostral Cingulate	100	-10, 44, 14	3.61	0.038

Supplementary Table 3: MNI peak coordinates of brain regions with Reward Prediction Error (Panel A) and Punishment Prediction Error (Panel B) in the Healthy Control (N = 26) and MDD (N = 25) group, modeled with individual learning rate; Identical initial cluster forming threshold of p = 0.005 and extent threshold of 60 voxels were used for easy visualization. [Note: To facilitate comparisons between analyses, clusters that were FWE significant in the fixed learning rate analyses are listed here even though they did not survive cluster correction in this analysis]

A. Reward Prediction Error

**Brain Region**	**Cluster size**	**MNI (x, y, z)**	**Z score**	**Cluster p (FWE)**
Healthy Controls				
Right Visual Cortex	6770	28, -78, -10	6.88	0.000
Right Calcarine Cortex	369	-14, -68, 10	4.76	0.000
Left Superior Parietal Cortex	140	-32, -42, 44	4.44	0.003
Right Anterior Insula	84	30, 28, -2	3.44	0.071
Right Putamen/NAc	64	14, 10, -10	3.99	0.238
MDD				
Left Visual Cortex	8551	-26, -74, -12	6.59	0.000
Left Inferior Frontal Gyrus	132	-46, 8, 24	4.79	0.007
Left Superior Parietal Cortex	87	-32, -48, 54	4.91	0.075
Supplementary Cortex	113	-4, 4, 66	4.15	0.018
Posterior Cingulate	87	2, -34, 30	3.77	0.075
Right Precentral Gyrus	118	32, 4, 24	3.53	0.014

B. Punishment Prediction Error

Brain Region	Cluster size	MNI (x, y, z)	Z score	Cluster p (FWE)
Healthy Controls				
Right Visual Cortex	3197	34, -64, -14	6.01	0.000
Left Visual Cortex	1838	-26, -88, 8	5.76	0.000
Left Fusiform Gyrus	2719	-26, -68, -14	5.51	0.000
Thalamus/Habenula (part of fusiform gyrus cluster)	113	2, -30, -2	4.74	0.000
Midcingulate	1520	6, 26, 30	5.05	0.000
Right Middle Frontal Gyrus	67	42, 8, 40	3.79	0.272
Right Anterior Insula	163	44, 12, 0	4.26	0.002
Right Anterior Insula	72	36, 18, -8	3.49	0.208
Left Precentral Gyrus	98	-42, 4, 26	3.97	0.050
Left Anterior Insula	798	-32, 24, 6	4.99	0.000
Left supramarginal gyrus	80	-52, -34, 34	3.84	0.134
MDD				
Left Visual Cortex	5641	-26, -80, 14	5.87	0.000
Right Fusiform Gyrus	4514	34, -78, -12	6.33	0.000
Thalamus/Habenula (part of fusiform gyrus cluster)	193	6, -24, 2	4.31	0.000
Midcingulate	1560	-6, 16, 50	5.00	0.000
Left Anterior Insula	722	-32, 26, 0	5.22	0.000
Right Inferior Frontal Gyrus	693	34, 30, 4	4.87	0.000
Right Middle Frontal Gyrus	437	44, 14, 28	5.51	0.000
Left Middle Frontal Gyrus	72	-36, 24, 22	4.21	0.166
Left Precentral Gyrus	508	-52, -4, 48	4.51	0.000

Right Supramarginal Gyrus	103	52, -30, 50	4.09	0.028
Rostral Cingulate	148	-10, 44, 14	4.16	0.003

REFERENCES

Chase HW, Kumar P, Eickhoff SB, Dombrovski AY (2015). Reinforcement learning models and their neural correlates: An activation likelihood estimation meta-analysis. *Cogn Affect Behav Neurosci* **15**: 435–459.

Chen C, Takahashi T, Nakagawa S, Inoue T, Kusumi I (2015). Reinforcement learning in depression: A review of computational research. *Neurosci Biobehav Rev* **55**: 247–267.

Daw ND (2011). Trial-by-trial data analysis using computational models. *Decision making, Affect and Learning: Attention and Performance XXIII:* 3–38.

Deichmann R, Gottfried JA, Hutton C, Turner R (2003). Optimized EPI for fMRI studies of the orbitofrontal cortex. **19**: 430–441.

Estes WK, Maddox WT (2005). Risks of drawing inferences about cognitive processes from model fits to individual versus average performance. *Psychon Bull Rev* **12**: 403–408.

First MB, Spitzer RL, Miriam G, Williams JB. (Biometrics Research, New York State Psychiatric Institute: New York, 2002). *Structured Clinical Interview for DSM-IV-TR Axis I Disorders, Research Version, Non-patient Edition. (SCID-I/NP)*.

Friston KJ, Buechel C, Fink GR, Morris J, Rolls E, Dolan RJ (1997). Psychophysiological and modulatory interactions in neuroimaging. *Neuroimage* **6**: 218–229.

Garrison J, Erdeniz B, Done J (2013). Prediction error in reinforcement learning: a meta-analysis of neuroimaging studies. *Neurosci Biobehav Rev* **37**: 1297–310.

Gradin VB, Kumar P, Waiter G, Ahearn T, Stickle C, Milders M, *et al* (2011). Expected value and prediction error abnormalities in depression and schizophrenia. *Brain* **134**: 1751–1764.

Hamilton M (1980). Rating depressive patients. *J Clin Psychiatry* **41**: 21–4.

Hennigan K, D'Ardenne K, McClure SM (2015). Distinct Midbrain and Habenula Pathways Are Involved in Processing Aversive Events in Humans. *J Neurosci* **35**: 198–208.

Kumar P, Waiter G, Ahearn T, Milders M, Reid I, Steele JD (2008). Abnormal temporal difference reward-learning signals in major depression. *Brain* **131**: 2084–2093.

Lawson RP, Drevets WC, Roiser JP (2013). Defining the habenula in human neuroimaging studies. *Neuroimage* **64**: 722–727.

Lawson RP, Seymour B, Loh E, Lutti A, Dolan RJ, Dayan P, *et al* (2014). The habenula encodes

negative motivational value associated with primary punishment in humans. *Proc Natl Acad Sci USA* **111**: 11858–63.

McLaren DG, Ries ML, Xu G, Johnson SC (2012). A generalized form of context-dependent psychophysiological interactions (gPPI): A comparison to standard approaches. *Neuroimage* **61**: 1277–1286.

Murty VP, Shermohammed M, Smith DV, Carter RM, Huettel SA, Adcock RA (2014). Resting state networks distinguish human ventral tegmental area from substantia nigra. *Neuroimage* **100**: 580–589.

Pessiglione M, Seymour B, Flandin G, Dolan RJ, Frith CD (2006). Dopamine-dependent prediction errors underpin reward-seeking behaviour in humans. *Nature* **442**: 1042–1045.

Salas R, Baldwin P, Biasi M de, Montague PR (2010). BOLD Responses to Negative Reward Prediction Errors in Human Habenula. *Front Hum Neurosci* **4**: 36.

Sutton RS, Barto AG (1998). Reinforcement learning: an introduction. *IEEE Trans Neural Netw* **9**: 1054.

Wilson RC, Niv Y (2015). Is Model Fitting Necessary for Model-Based fMRI? *PLOS Comput Biol* **11**: e1004237.
