# Supplementary material for: Impaired reward prediction error encoding and striatal-midbrain connectivity in depression
Source: Neuropsychopharmacology. 2018 Feb 26;43(7):1581–8. doi: 10.1038/s41386-018-0032-x (PMC5983542; doi:10.1038/s41386-018-0032-x)
Supplement: Supplementary file 2 — Supplementary Figures(PDF 2498 kb) [file 41386_2018_32_MOESM2_ESM.pdf]

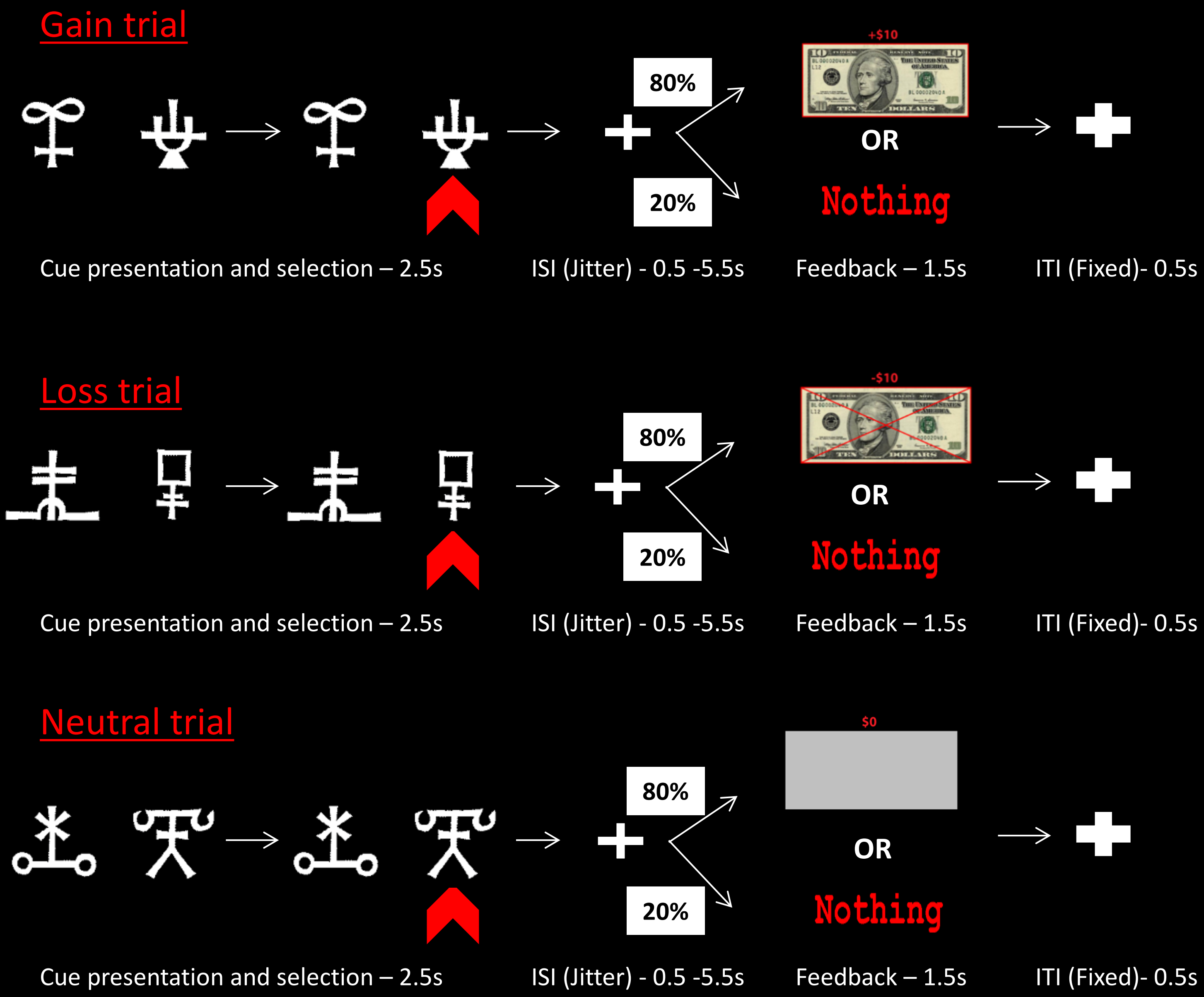
Gain trial
80%
20%
+$10
OR
Nothing
Cue presentation and selection – 2.5s
ISI (Jitter) - 0.5 -5.5s
Feedback – 1.5s
ITI (Fixed)- 0.5s
Loss trial
80%
20%
-$10
OR
Nothing
Cue presentation and selection – 2.5s
ISI (Jitter) - 0.5 -5.5s
Feedback – 1.5s
ITI (Fixed)- 0.5s
Neutral trial
80%
20%
$0
OR
Nothing
Cue presentation and selection – 2.5s
ISI (Jitter) - 0.5 -5.5s
Feedback – 1.5s
ITI (Fixed)- 0.5s

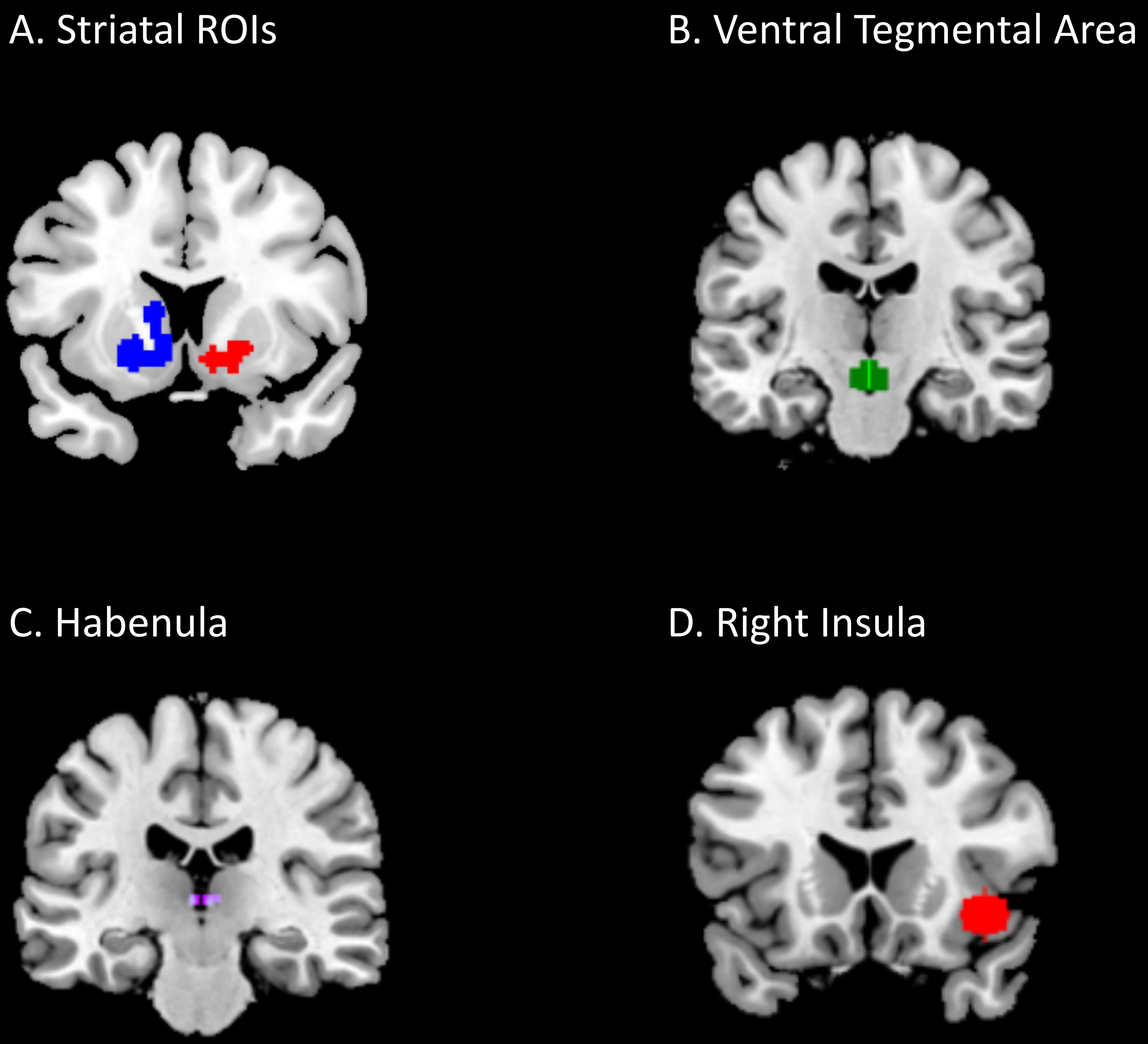
A. Striatal ROIs
B. Ventral Tegmental Area
C. Habenula
D. Right Insula

A. Reward Prediction Error – Healthy Controls
Group Fixed Learning Rate

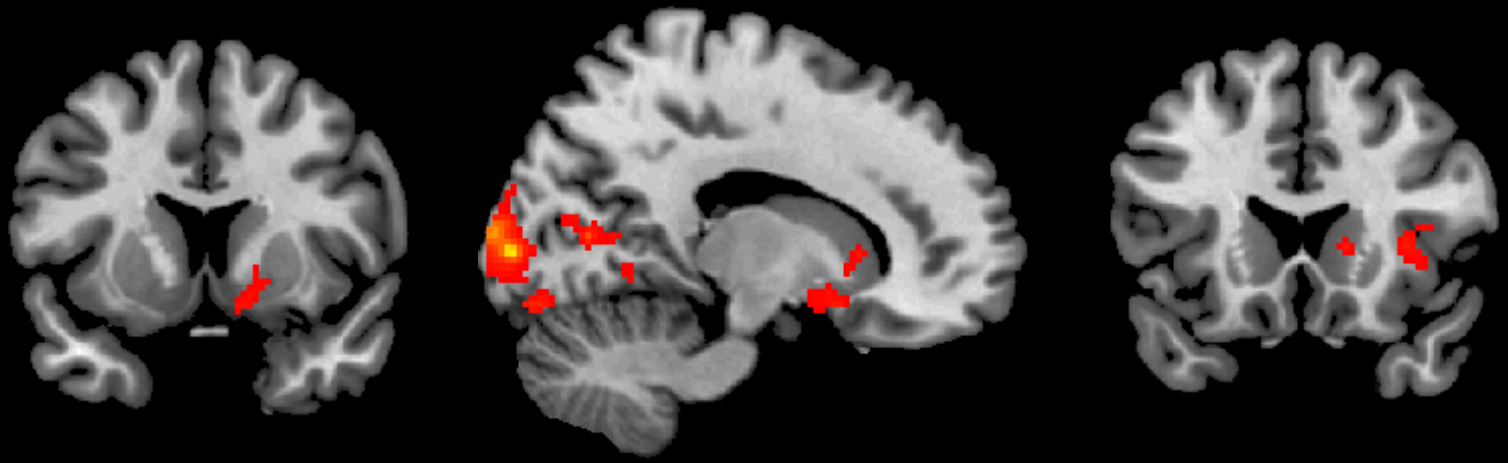

B. Reward Prediction Error – MDD
Group Fixed Learning Rate

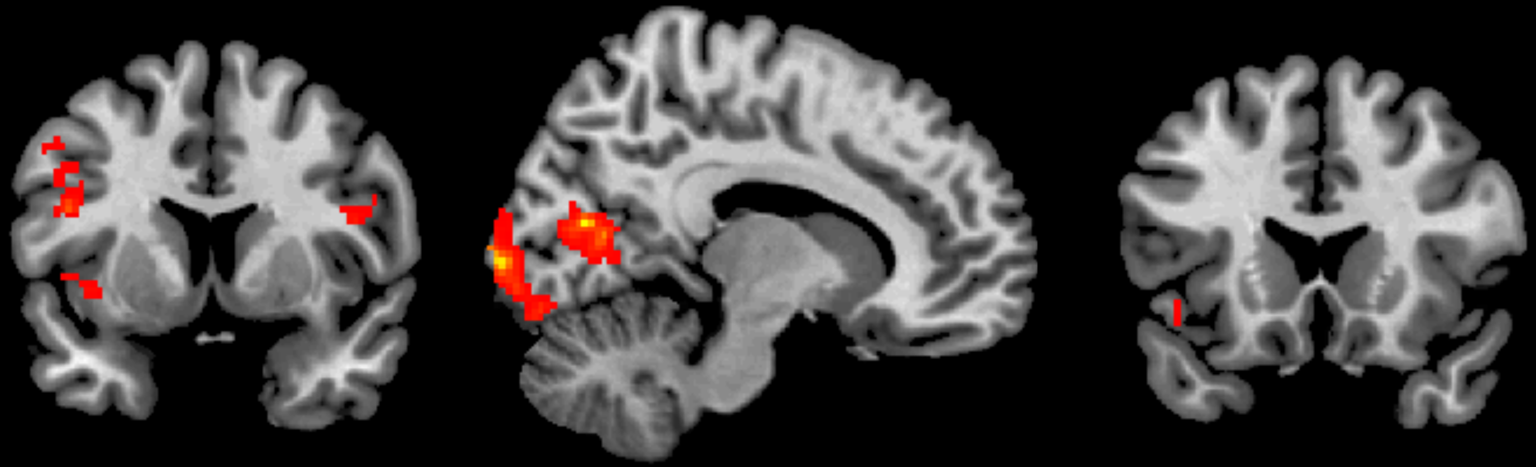

A. Punishment Prediction Error – Healthy Controls
Group Fixed Learning Rate

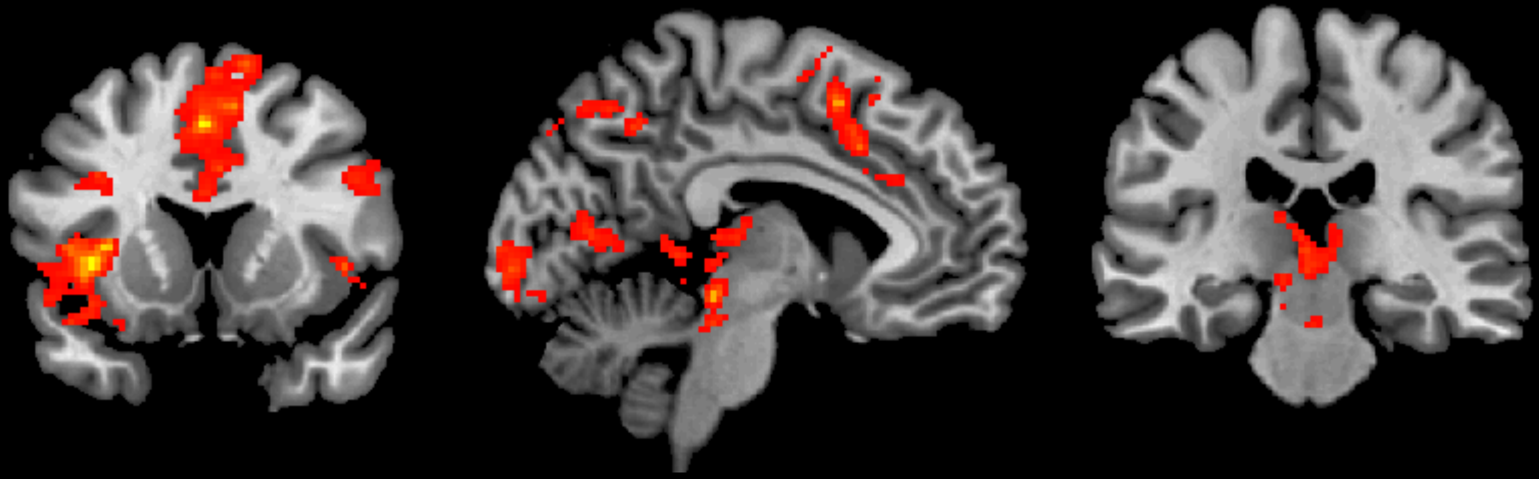

B. Punishment Prediction Error – MDD
Group Fixed Learning Rate

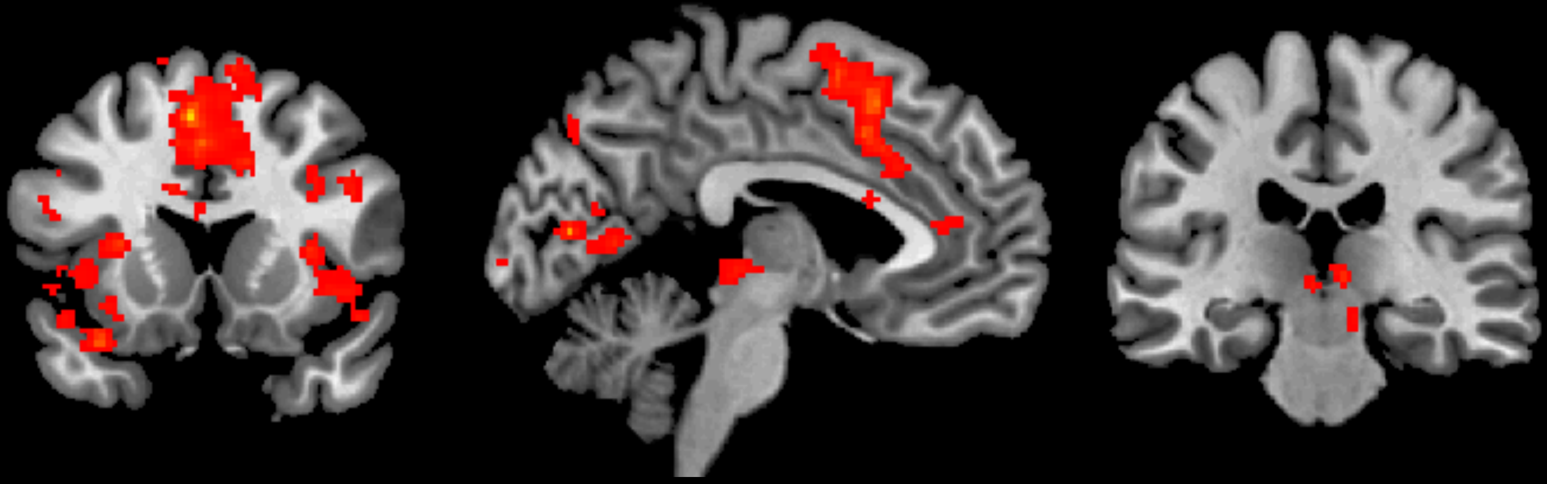

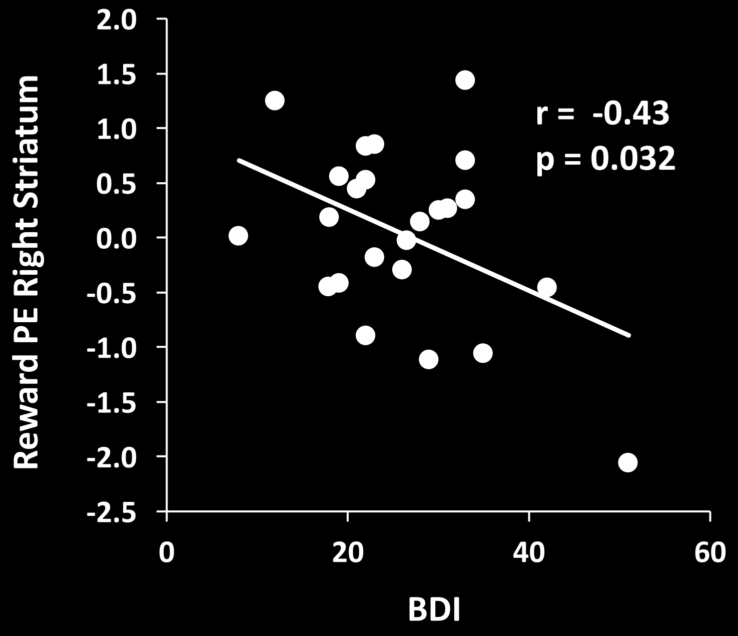

Reward PE Right Striatum
2.0
1.5
1.0
0.5
0.0
-0.5
-1.0
-1.5
-2.0
-2.5
0
20
40
60
BDI
r = -0.43
p = 0.032

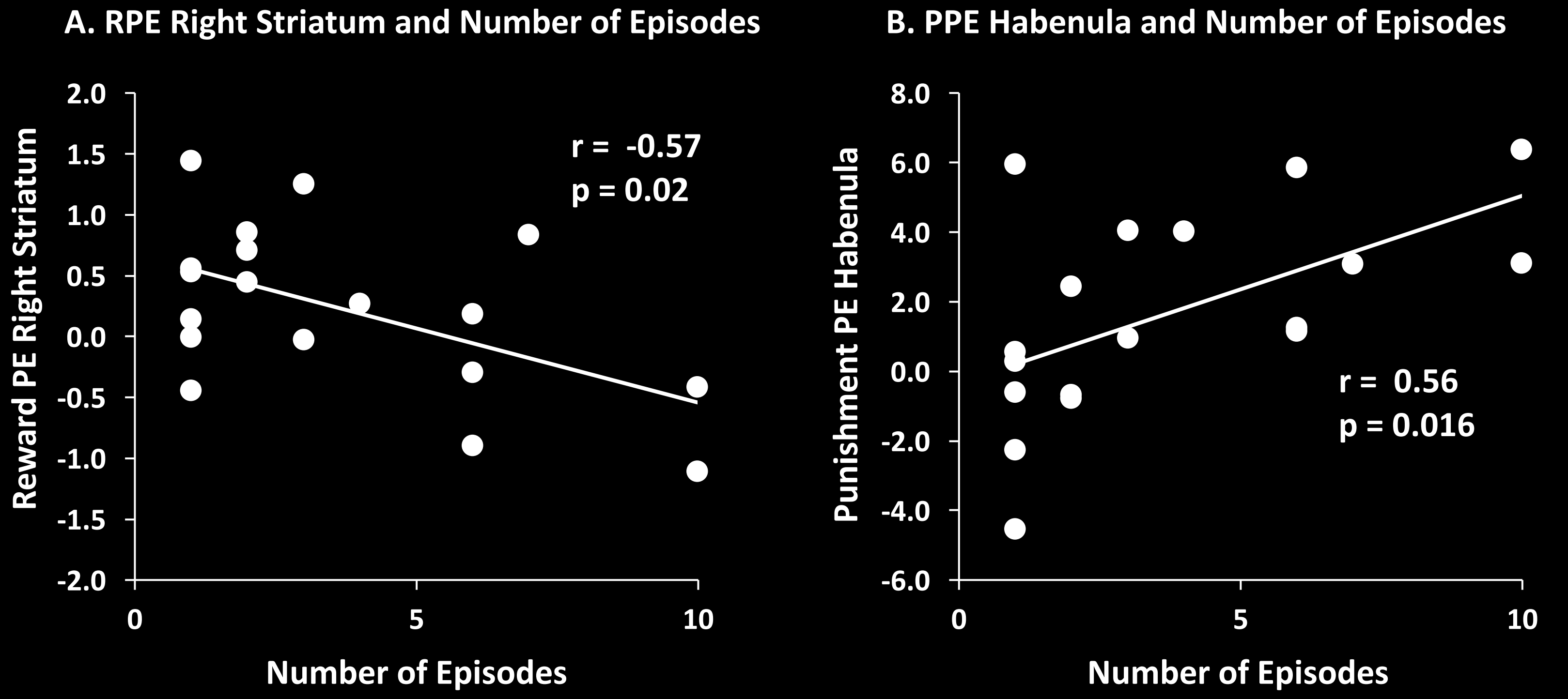
A. RPE Right Striatum and Number of Episodes
Reward PE Right Striatum
2.0
1.5
1.0
0.5
0.0
-0.5
-1.0
-1.5
-2.0
0
5
10
Number of Episodes
r = -0.57
p = 0.02
B. PPE Habenula and Number of Episodes
Punishment PE Habenula
8.0
6.0
4.0
2.0
0.0
-2.0
-4.0
-6.0
0
5
10
Number of Episodes
r = 0.56
p = 0.016

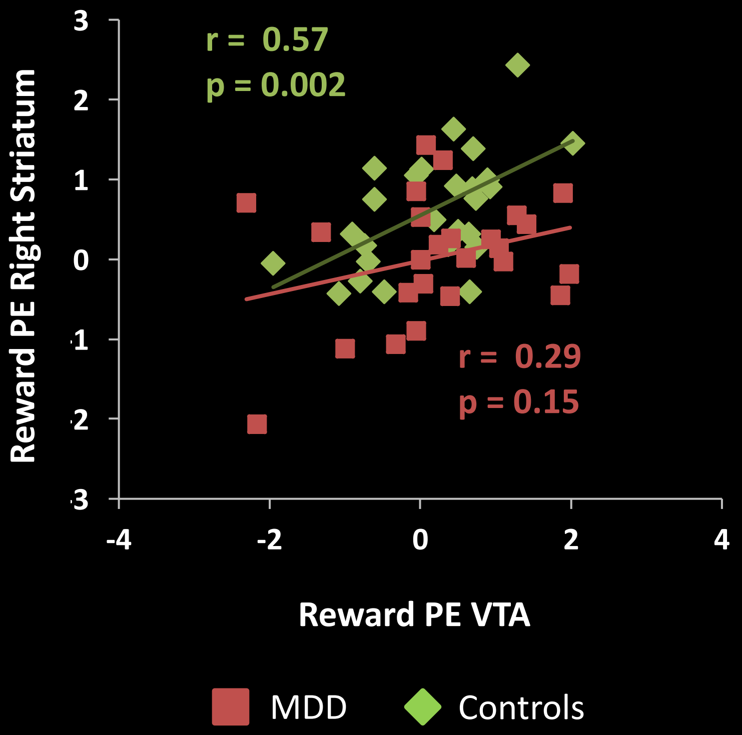
r = 0.57
p = 0.002
r = 0.29
p = 0.15
Reward PE Right Striatum
Reward PE VTA
3
2
1
0
-1
-2
-3
-4
-2
0
2
4
MDD
Controls

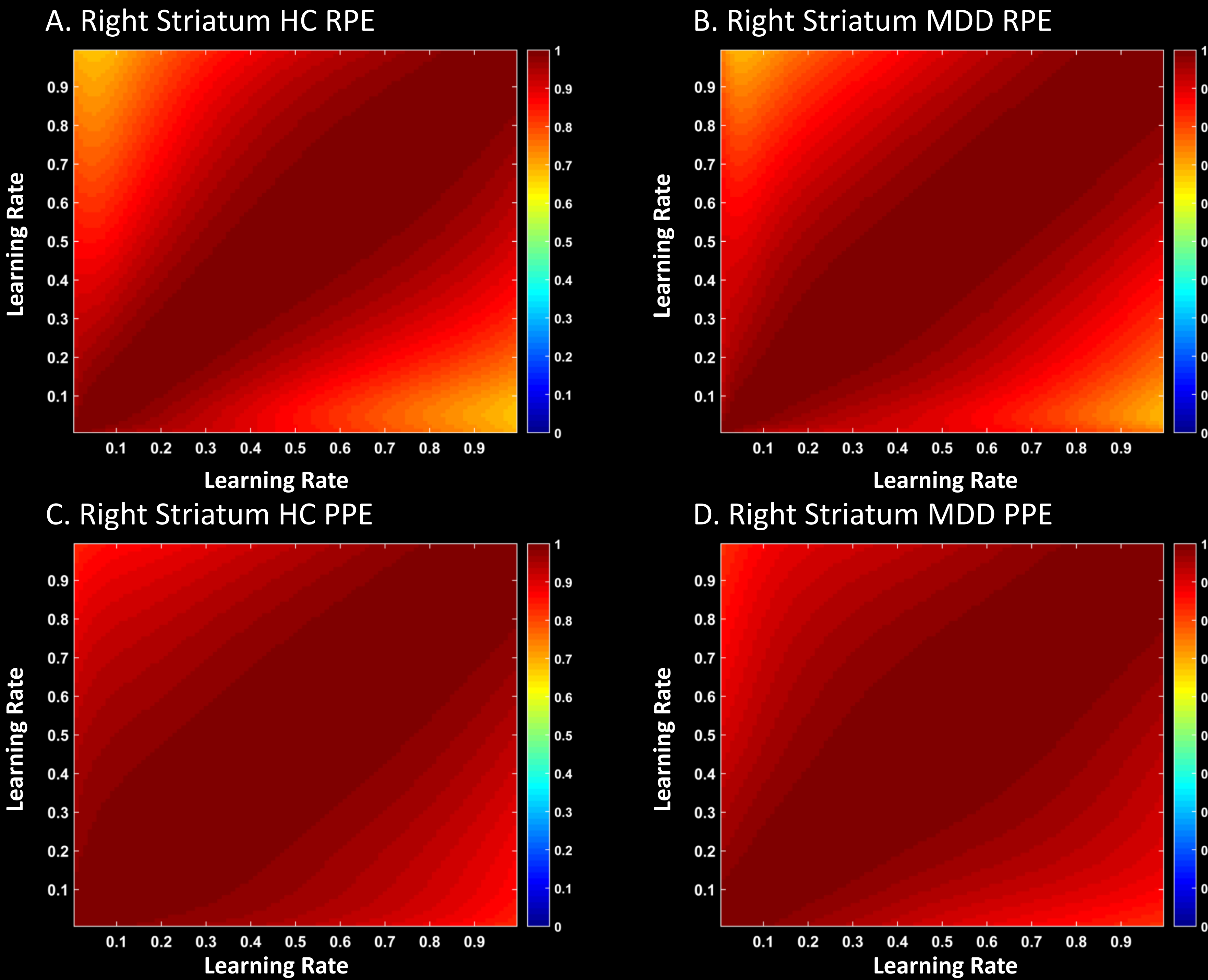

A. Right Striatum HC RPE
B. Right Striatum MDD RPE
C. Right Striatum HC PPE
D. Right Striatum MDD PPE
Learning Rate
0.1
0.2
0.3
0.4
0.5
0.6
0.7
0.8
0.9
0
1

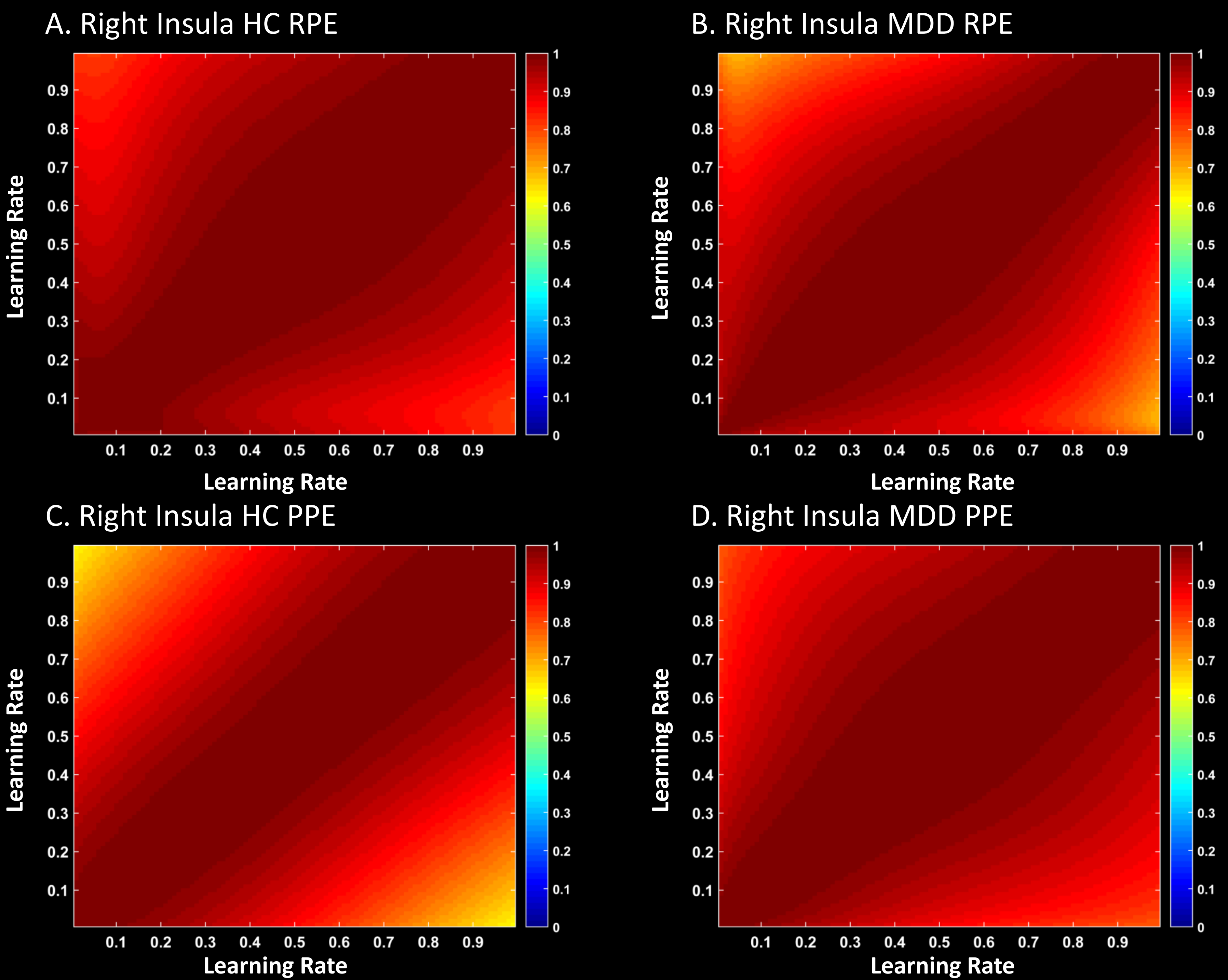

A. Right Insula HC RPE
B. Right Insula MDD RPE
C. Right Insula HC PPE
D. Right Insula MDD PPE
Learning Rate
Learning Rate
0.1 0.2 0.3 0.4 0.5 0.6 0.7 0.8 0.9
0 0.1 0.2 0.3 0.4 0.5 0.6 0.7 0.8 0.9 1

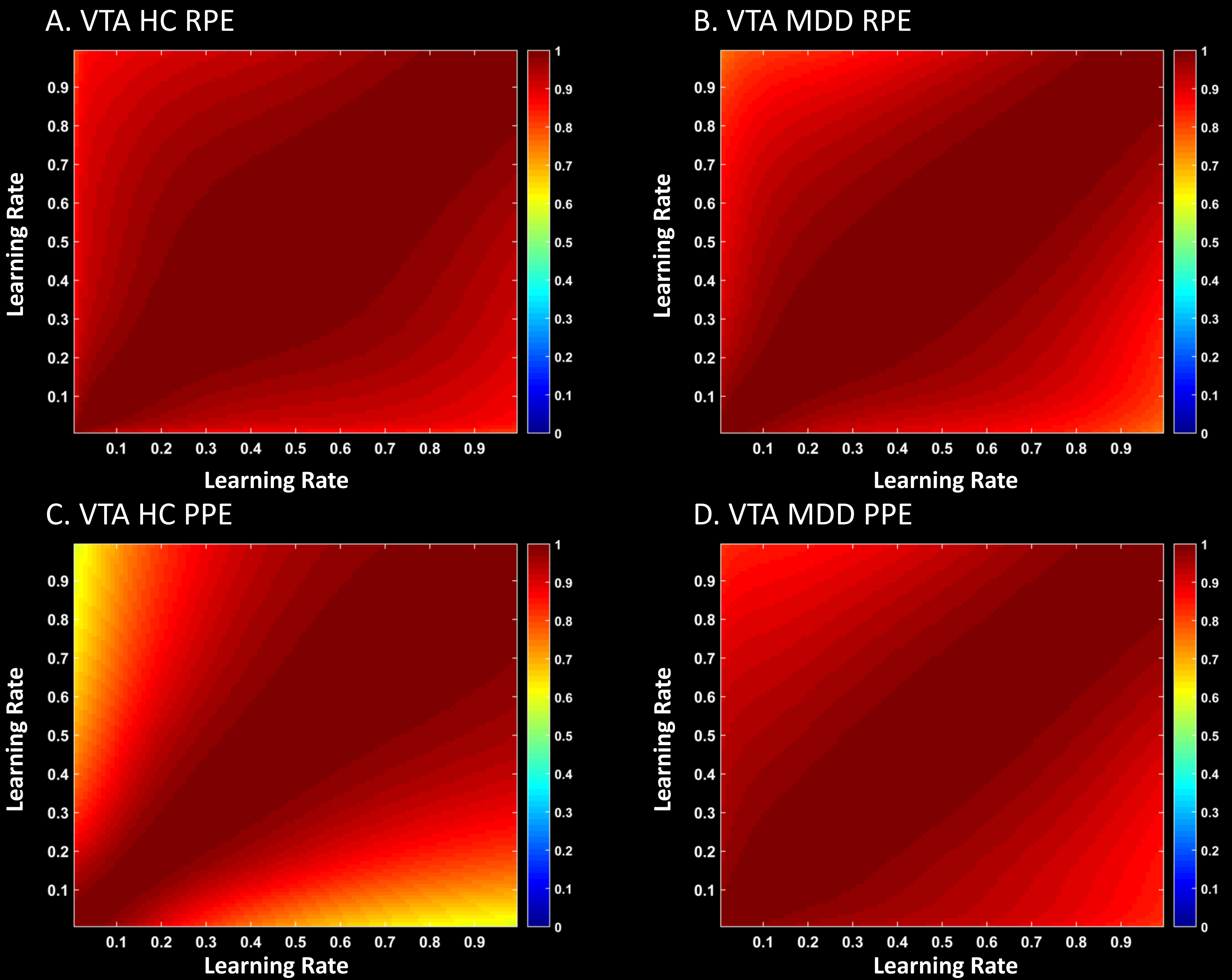

A. VTA HC RPE
B. VTA MDD RPE
C. VTA HC PPE
D. VTA MDD PPE
Learning Rate
Learning Rate
0.1
0.2
0.3
0.4
0.5
0.6
0.7
0.8
0.9
0
1

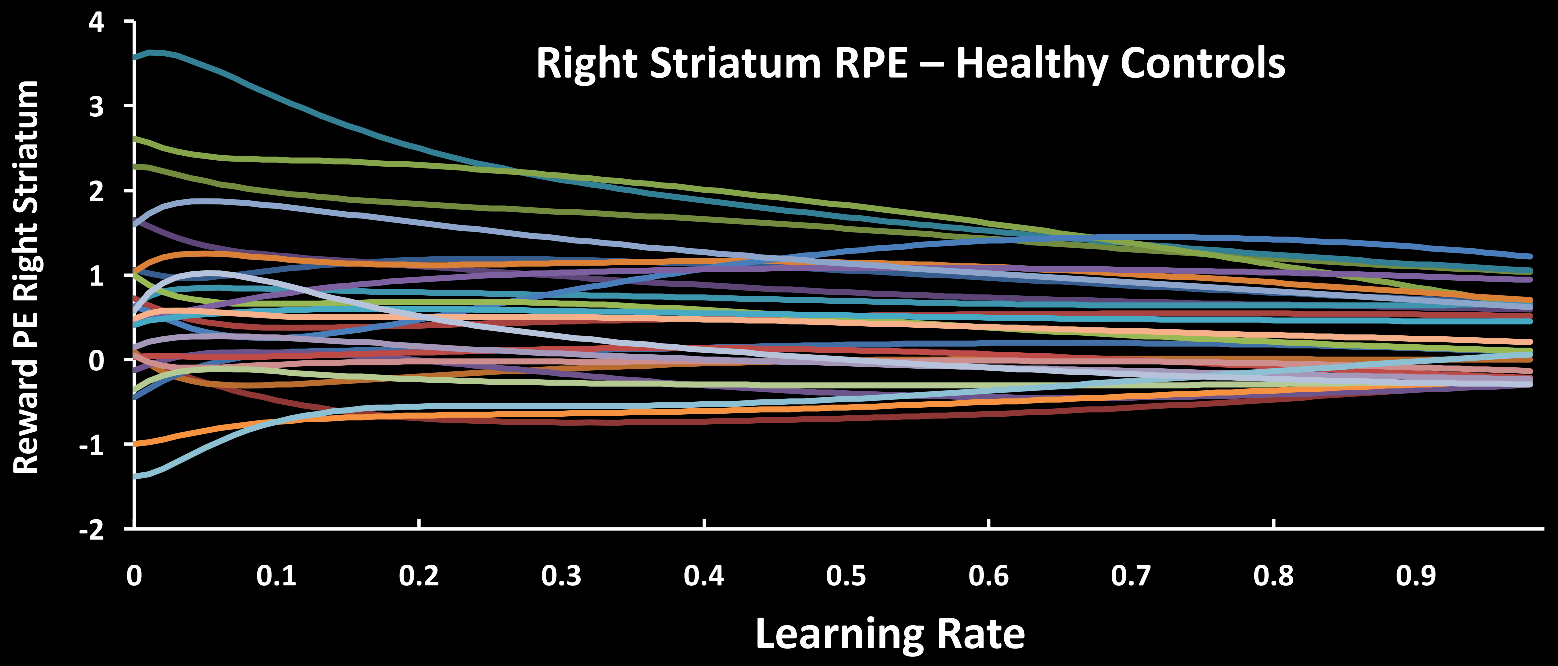
Right Striatum RPE – Healthy Controls
Reward PE Right Striatum
4
3
2
1
0
-1
-2
0
0.1
0.2
0.3
0.4
0.5
0.6
0.7
0.8
0.9
Learning Rate

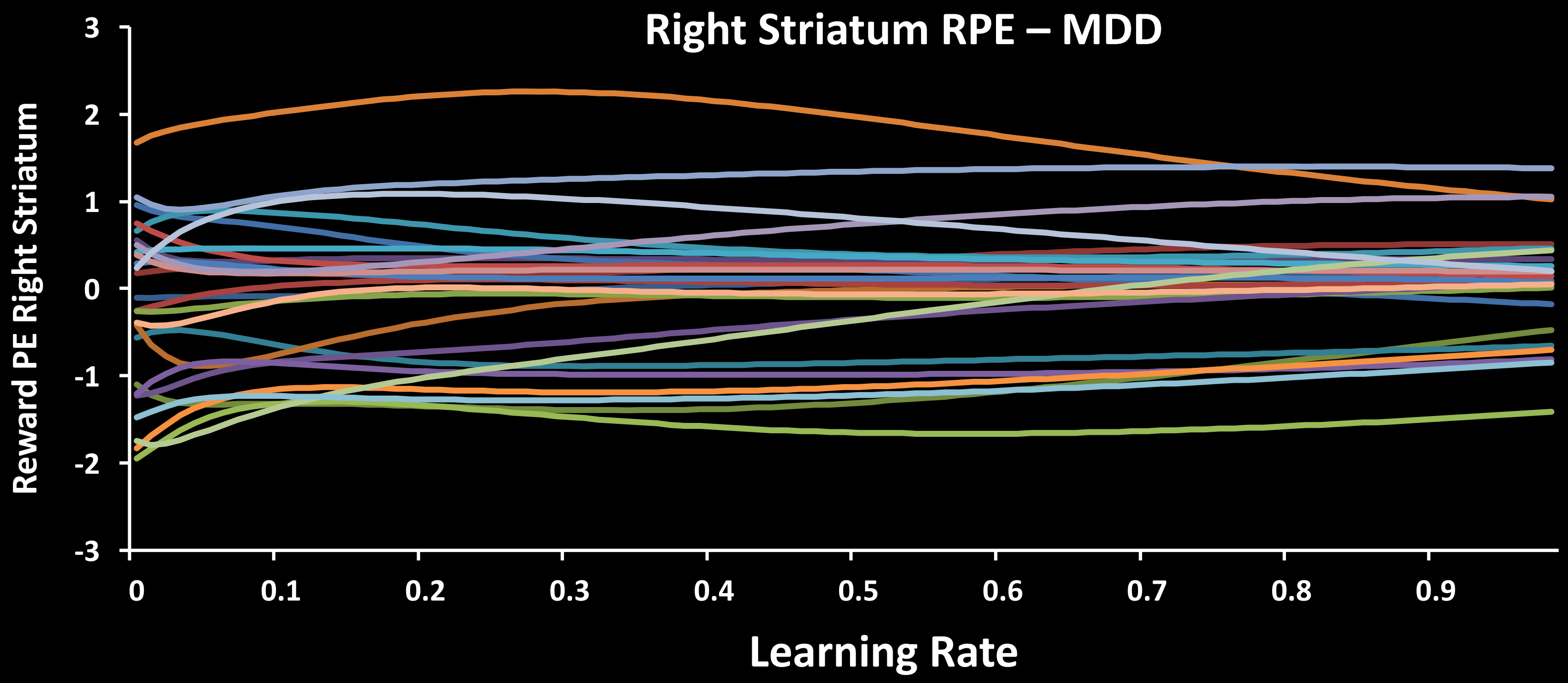
Right Striatum RPE – MDD
Reward PE Right Striatum
3
2
1
0
-1
-2
-3
0
0.1
0.2
0.3
0.4
0.5
0.6
0.7
0.8
0.9
Learning Rate

A. Reward Prediction Error – Healthy Controls
Individual Learning Rate

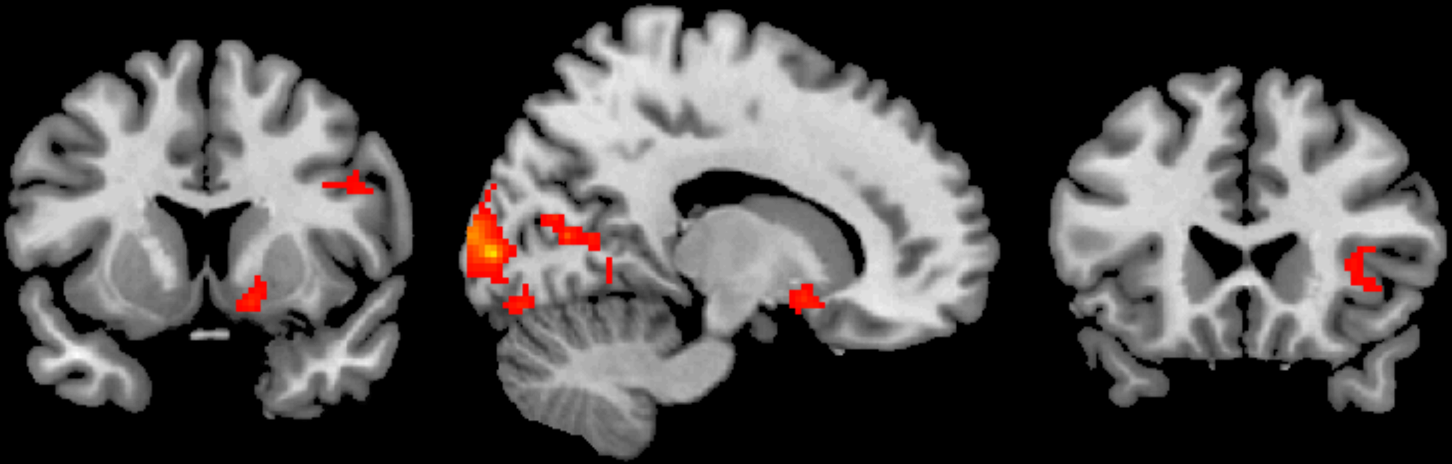

B. Punishment Prediction Error – Healthy Controls
Individual Learning Rate

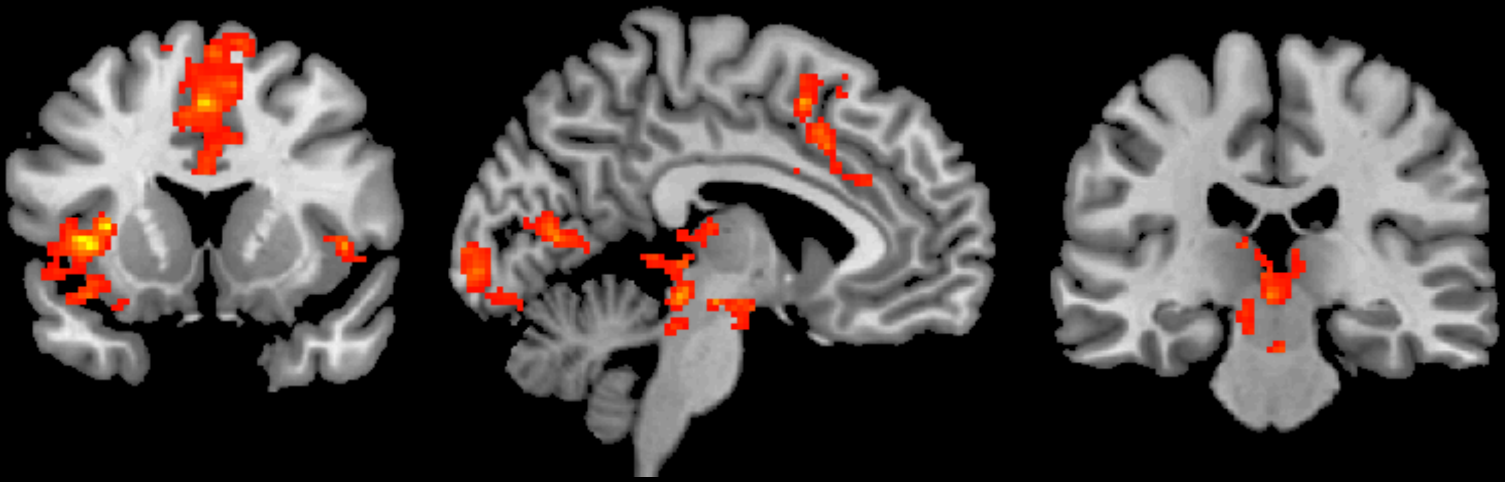

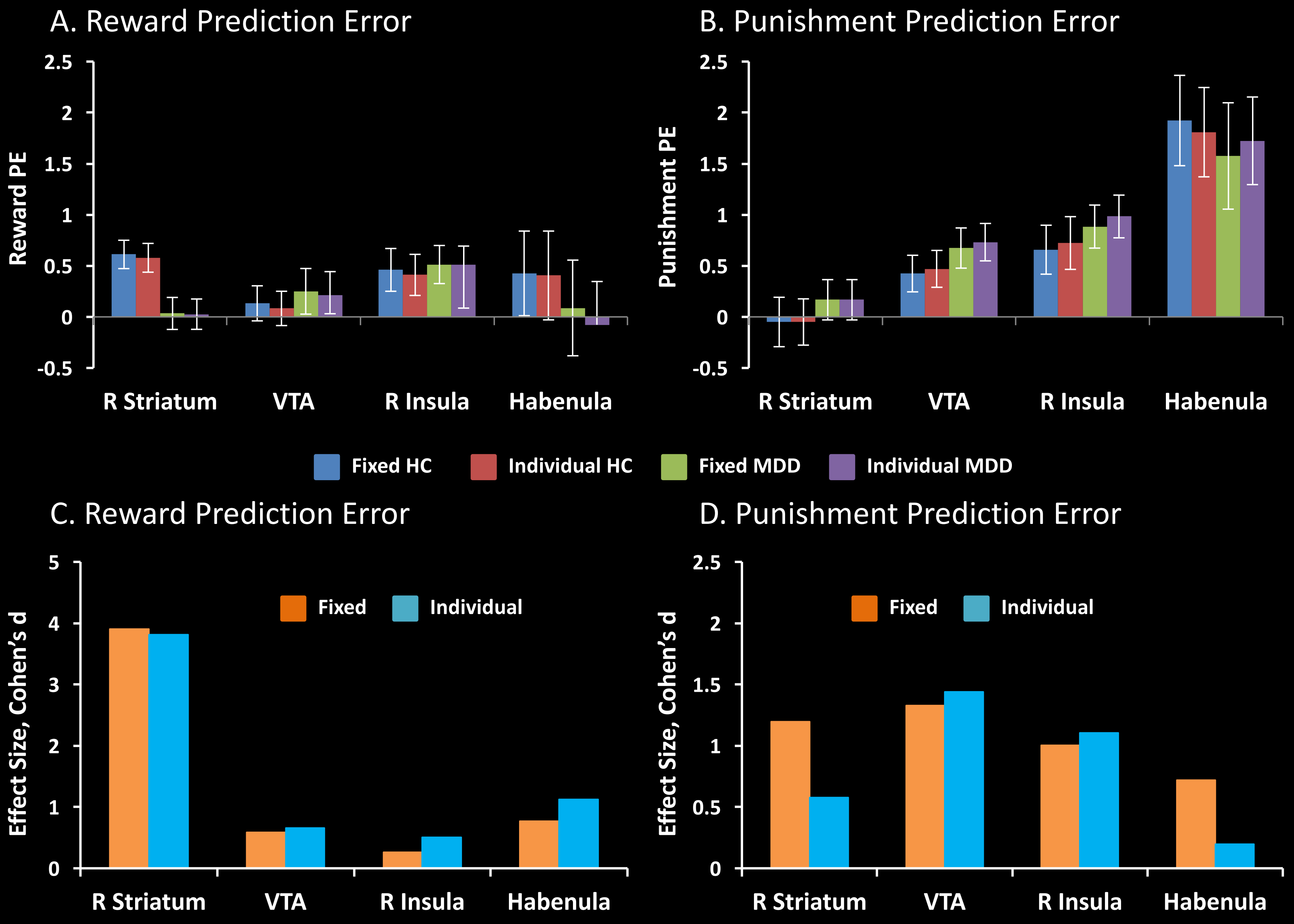

A. Reward Prediction Error
B. Punishment Prediction Error
Reward PE
Punishment PE
2.5
2
1.5
1
0.5
0
-0.5
R Striatum
VTA
R Insula
Habenula
Fixed HC
Individual HC
Fixed MDD
Individual MDD
C. Reward Prediction Error
D. Punishment Prediction Error
Effect Size, Cohen's d
5
4
3
2
1
0
Fixed
Individual
